# Flexural strengthening of beams using post-tensioned metal straps fully wrapped around steel channels anchored to normal steel reinforced concrete beams

**DOI:** 10.1371/journal.pone.0253816

**Published:** 2021-06-30

**Authors:** Wrya Abdullah, Serwan Khwrshid Rafiq

**Affiliations:** Civil Engineering Department, College of Engineering, University of Sulaimani, Sulaimani, Kurdistan Region, Iraq; RMIT University, AUSTRALIA

## Abstract

The efficacy of post-tensioned metal straps PTMS, wrapped around steel channels anchored to normal reinforced concrete (R.C) beams is tested in increasing the flexural capacity of the beams. For this purpose, nine normal R.C beams with dimensions of 160 mm x 240 mm x 2100 mm are constructed to fail in bending. The location and the number of the straps are considered as the main variable. It is found that using PTMS can enhance the load-carrying capacity of the beam by 29% to 63%. The decisive factors affecting the increase are the location of the straps (at the bottom or sides), shape of the flange and web edges (squared or rounded) and alignment of the flanges (vertical or inclined). A complete guide can be found in the paper as it is a novel method of strengthening beams which can be applied to the beams cast in place with integral slabs.

## 1. Introduction

Reinforced concrete beams require strengthening to upgrade their strength, stiffness and ductility capacity which may be required due to design errors, needs for additional load carrying capacity or after an accidental load such as that of an earthquake. Strengthening with Post Tensioned Metal Straps (PTMS) is a novel technique for strengthening structural members developed by University of Sheffield in England and University of Patras in Greece for the first time in 1995 by Frangou et al. [1]. The target was to develop a method which is easier and more cost-effective especially as the other effective methods of strengthening are more expensive.

The method consists of using flexible and ultra-high strength steel with tensile yield strength of approximately 1000 MPa in shape of straps with various widths varying from 15 mm to 32 mm and the thickness of 0.5 to 1 mm. The straps will be wrapped around the members with a pneumatic tensioning machine to apply tensile stress to confine it and then sealed in shape of “push type” with mechanical clips with a sealer [2]. Therefore, the application consists of four essential elements namely pneumatic tension machine, sealer, clips and metal straps.

Many researchers have studied using PTMS to strengthen different elements in various ways both practically [1–21] and theoretically [17, 22–25]. Most of those studies were carried out in shape of testing small specimens such as cylinders with diameter of 150 mm and height of 300 mm [1, 4, 10, 26, 27]. While others used prisms of different sizes such as 100 mm * 100 mm * 200 mm which are depth, width and length respectively [28, 29]. To fully comprehend the method of PTMS, more research studies should be carried out. This will help in developing the design and retrofitting equations.

The main function of the material is to increase the confinement which is beneficial for short columns under lateral loads, beams failed in shear and for high-strength slender columns. However, for beams failed in flexure, the method of using it effectively has been one of the challenges, therefore, very few researchers studied it. C. L. Chin et al. [29] tested twelve square columns with the dimension of 100 mm^2^ and the height of 200 mm. They tested the samples in their squared shape. The compressive strength of used concrete was 68 MPa on its 28^th^ day of curing and it was 82 MPa on the day of the test. The tensile strength of the metal strap was 925 MPa on average. Ten of the twelve samples were confined using PTMS and a pair of those confined was tested using five parameter variables. The number of layers was varying between two, three, and four and the spacing between the straps was varying from 10 mm to 30 mm with 20 mm in between. They inferred that with increasing the layers of PTMS and reducing the spacing, the compressive strength increases by up to 31% as it was the case for the sample strengthened with 4 layers with 10 mm of spacing. They, also, reported an increase of an approximately 137% in deformation for samples strengthened with 3 layers with spacing of 10 mm.

Their results may vary for larger column specimens reinforced with steel bars. Also, for each variable parameter, they had two samples which had various unexplained differences between the identical pairs. For example, for the samples confined with 3 layers of PTMS with spacing of 10 mm, the deformation capacity increased by 78% to 137% while the same deformation capacity for 4 layers with 10 mm spacing was 77% to 111%. Setkit and T. Imjai [4] tested beams failed in flexure, however, they used fully wrapped metal straps as a confinement of the beams to enhance flexural strength of the beam, therefore, the results of confined and unconfined of the beams are nearly the same but only the ductility is improved.

For this reason, it is necessary to develop the method to be used for beams failed in flexure which is the main purpose of this paper. The conventional use of PTMS is to increase the lateral confinement. This purpose is achieved by wrapping PTMS independently around the elements. However, to knowledge of the authors, this is the first that PTMS is being used with steel channels to enhance the load carrying capacity of R.C beams. The method is practical and can be used on R.C beams in any frame building.

## 2. Experimental programme

**Fig 1:**
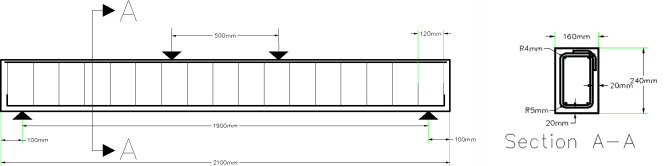
Beam specimen details. (a) Layout of the beam. (b) Cross-section of the beam.

Nine normal reinforced concrete (R.C) beams are cast and tested using four-point loading. The clear span of beams was 1900 mm with another 200 mm left for the supports. So, the length of the specimens was 2100 mm. The cross- section of the beams is 160 mm x 240 mm which are width and depth respectively. The steel reinforcement details of the beams are shown in Fig 1(A) and 1(B). The beam has two main reinforcement bars. The diameter of bars is 10 mm with a vertical hook of 70 mm at the end of each rebar. It has two reinforcement bars at top of the beam. The top reinforcement diameter is 8 mm. The diameter of the stirrups is 10 mm with a spacing of 105 mm based on the design for maximum spacing which is controlled by *d*/2 where *d* is the effective depth of the beam. The stirrups are provided to ensure the flexural failure of the beams.

The concrete casting is done in four different days with a day between each of the four using four different concrete mixes. Four cylinders are taken from each specimen mix to measure the compressive strength and the split tensile strength of the concrete for each of the beams. The average compressive strength of the beams is found to be 27.4 MPa and the tensile strength of the beam is found to be 3.4 MPa using split tensile test. The slump of the concrete is 110 mm.

A beam is designated as a control beam and tested without any strengthening. The rest of the beams are strengthened to increase the load-carrying capacity of the beams. Strengthening is performed using metal straps wrapped around two steel channels and tensioned longitudinally using a pneumatic tensioner.

### 2.1 Materials and specimen fabrication

The mix design was chosen to achieve a characteristic compressive strength of 25 MPa as this is typical grade of concrete used in Sulaimania. Ordinary Portland Cement with IQS 5-CEM I 42.5 R is used which is commercially available with Mass Company, Sulaimania, Iraq. The source of the used coarse aggregate is mixture between the natural and crushed stones from a quarry in Piramagrun, Sulaimania city, Kurdistan Region, Iraq. The maximum size of the coarse aggregate is 10 mm. The data of its sieve analysis complies with that of ASTM C33 [30]. The other properties of the coarse aggregate are measured and shown in Table 1.

**Table 1:** Properties of course aggregate.

Properties of course aggregate	Value
**Bulk dry specific gravity**	2.632
**Bulk saturated surface dry specific gravity**	2.667
**Apparent specific gravity**	2.729
**Water absorption**	1.364%
**Dry density**	1480.8 kg/m^3^
**Compacted dry density**	1612.77 kg/m^3^

Additionally, the source of the fine aggregate was natural river sand from the same quarry in Piramagrun, Sulaimania, Kurdistan Region, Iraq. The data of its sieve analysis complies with ASTM C33 [30]. The properties of fine aggregate are listed in Table 2.

**Table 2:** Properties of fine aggregate.

Properties of fine aggregate	Value
**Bulk dry specific gravity**	2.571
**Bulk saturated surface dry specific gravity**	2.618
**Apparent specific gravity**	2.698
**Water absorption**	1.833%
**Dry density**	1509 kg/m^3^
**Compacted dry density**	1658.57 kg/^m3^

The water used in all the mixes is clean and drinkable water from a well next to the laboratory. To construct the reinforced beams, steel bars of diameters of 10 mm from Mass Company in Sulaimania city, Iraq, are used. The tensile strength of them are shown in Table 3.

**Table 3:** Properties of steel bars.

Specimen number	Yield Strength (MPa)	Ultimate strength (MPa)	Elongation %
**1**	504.8	640.7	22.5
**2**	504.9	635.9	27
**3**	502.3	638.1	27
**Average**	504	638.23	25.5

The concrete mix is designed based on ACI 211.1–91. The mixing ratio of 1: 2.02: 2.384 is used between cement, coarse aggregate, and fine aggregate respectively. The water to cement ratio of 0.61 is used in the mix. Table 4 shows the mix proportion of concrete.

**Table 4:** Mass of the ingredients of the concrete.

Water (kg)	Cement (kg)	Coarse aggregate (kg)	Fine aggregate (kg)	Total(kg)
**255**	380	769	907	2311

Two strain gauges are attached to the two main rebars at the bottom of the beam to measure the strain in the tension zone inside the concrete beam. To assure the concrete cover, circular plastic chairs of diameters of 25 mm are wrapped around the steel bars before casting. The specimens were cured continuously for 28 days.

### 2.2 Strengthening

**Fig 2:**
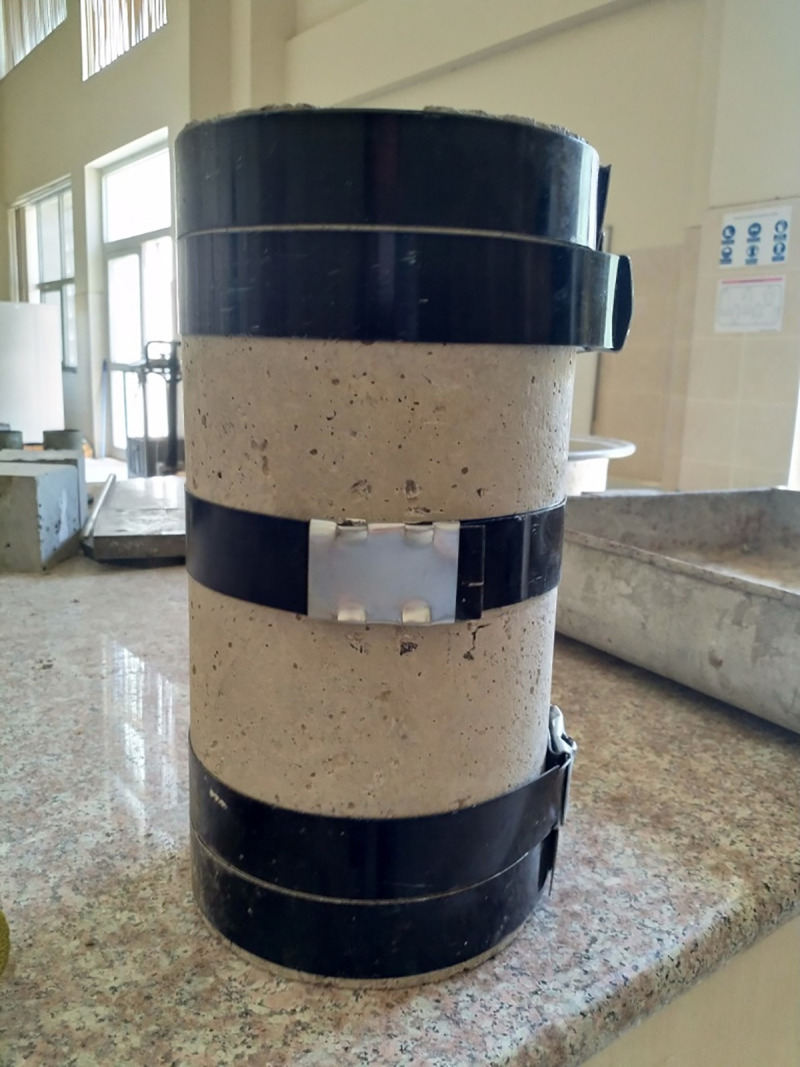
Metal strap and a clip.

For strengthening, the following materials are used. First, heavy duty metal straps from Jiangsu Juhong Strapping Manufacturing Company, China are used for strengthening of the beams. They are tested based on ASTM D3953 [31]. The material has a width of 31.75 mm and a thickness of 0.8 mm giving it a cross-sectional area of 25.4 mm^2^. An example of the metal strap with the clip is shown in Fig 2. To lock the two ends of the metal straps together, aluminium clips are used.

To ensure the lock, 4 notches are created using jaws from a pneumatic sealer attached to the pneumatic tensioner. Relaxation is a one of the concerns in using PTMS as notches on the clips might loosen and cause relaxation loss. For this reason, Moghadam et al. [16] tested a setup attaching load cells to a data logger for two consecutive months to monitor relaxation loss in the straps. They proved that, using this method for confinement has no relaxation within this time.

The compositions of the metal strap material and the properties given by the manufacturer are shown in Table 5. The metal strap specimens are tested both in China giving the yield strength of 928 MPa and in Sako Engineering Bureau, Sulaimania, Iraq, giving the maximum strength of 913 MPa. The modulus of elasticity of the strap is found to be 237 GPa.

**Table 5:** Properties of the metal strap.

Batch	Actual dimensions (mm)	Surface coating	Tensile test		Chemical composition				
			Tensile strength (MPa)	Elongation	C	Mn	Si	P	S
**E191015-1**	32*0.8	Black painted and waxed	935	9%	0.16	0.63	0.16	0.029	0.011

**Fig 3:**
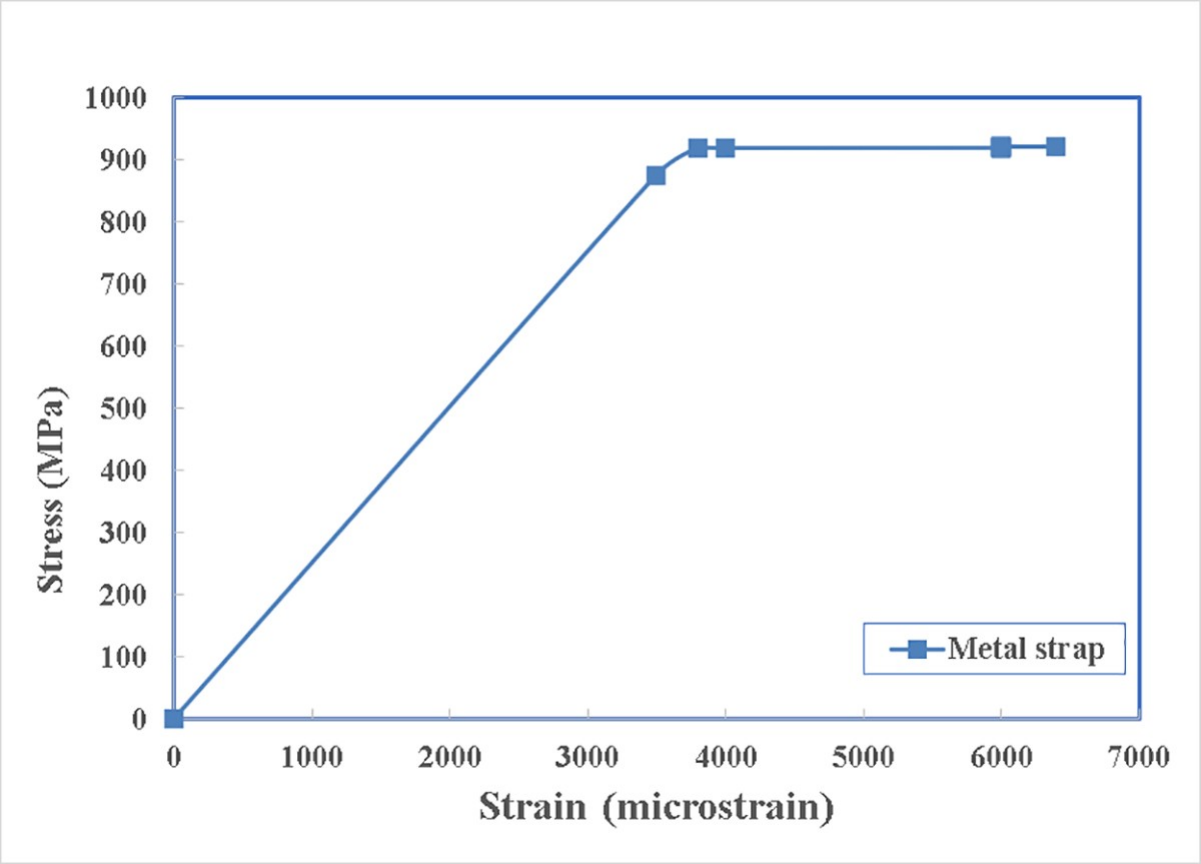
Manufacturer’s load- strain diagram of the metal strap.

The stress and strain of the specimen tested in China are shown in Fig 3. The behaviour of the material is nearly elastic perfect plastic so, where it reaches the yield strength with increasing the stress on it, it will stay there for an elongation of 9% until it reaches the ultimate elongation that corresponds to its ultimate strength which is equal to the yielding stress before its breakage.

For post tensioning the straps, pneumatic tensioner and sealer are needed. They are commercially available in the market of packaging separately and combined. The machine used is specifically designed to apply tension on the straps of a width of 32 mm. It consists of two parts which are the tensioning part and the sealer. They are connected to an air compressor using a special pneumatic hose. The product’s name is KZ-32 strap packing tools. The tensile capacity of the instrument is varying from 0.5–0.8 MPa. If an air pressure of 0.6 MPa applied to the instrument, it can deliver more than 9.8 kN. The strength of the sealing part is more than 18.4 kN.

It is important to remember that the work should be carried out in a clean environment otherwise the pneumatic tensioner and sealer might stop working as it runs only by the air from the air compressor. The capacity of the air compressor used is 160 litres with air pressure of 7 bars.

**Fig 4:**
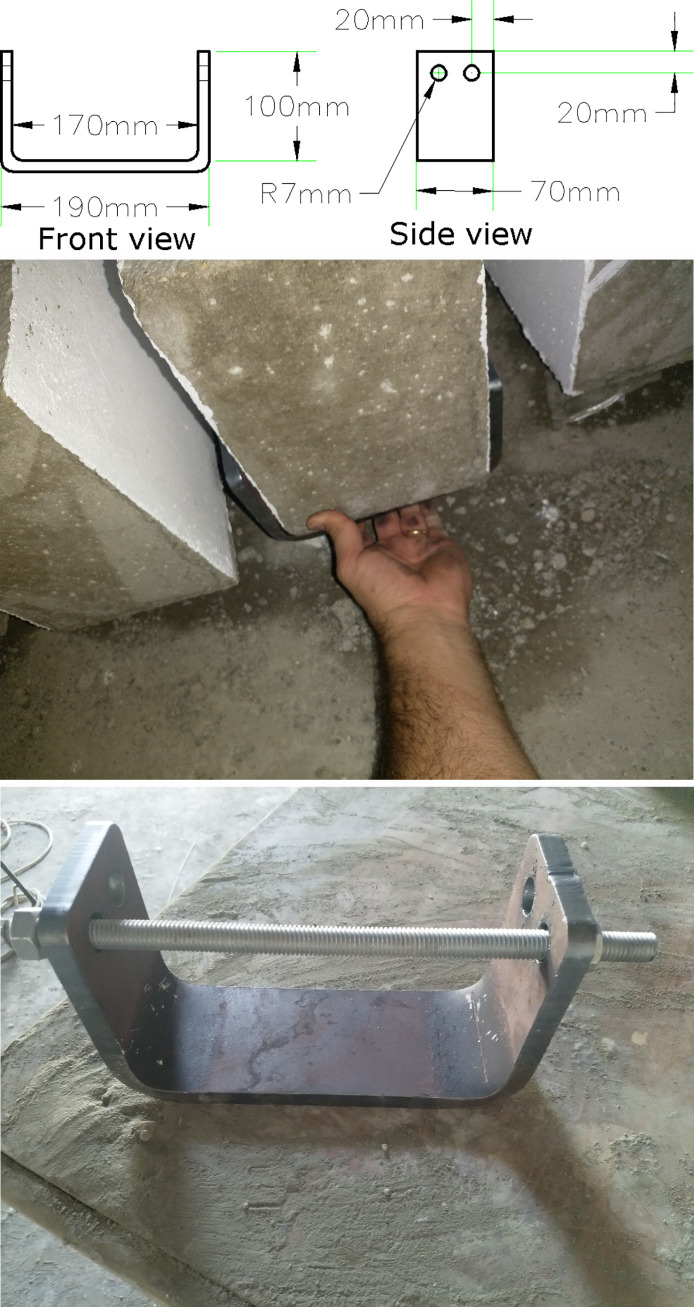
Steel channel details. (a) Front view and side view of the steel channel. (b) Steel channel in its location on the beam. (c) Steel channel with a full body stud bolt.

The straps will be wrapped around steel channels before post-tensioning. The steel channel is A36 steel with a thickness of 10 mm which is recommended by Garcia et al. [21]. The properties of the steel compiles with ASTM A36/A 36M [32]. The channel fits the width of the beam. Also, it will be anchored to the beam faces at the sides of the beam. Therefore, the clear width of the channel must be 170 mm based on several trials. This is to house the side straps safely and apply tension on them freely. The flange height is found to be 100 mm from the top face of the steel channel web to the highest point of the channel flange. This is to prevent having a bolt being aligned with the main steel reinforcement of the beam. The decisive factors in selecting the height of the flange are the number of the strap layers that might be used later in strengthening and the cover of the concrete which should be counted for during drilling holes. A stud detector is used to find the location of the stirrups inside the beam to avoid having holes in that vicinity. Once the channel is constructed in the steel factory and the flanges are bent in right angles, there is a rounded edge inside the channel that makes it impossible to have a full contact with the bottom face of the concrete beam. This space can be exploited to house metal straps at the bottom of the beam in the strengthening process. The dimensions of a channel are shown in Fig 4.

In Fig 4, there are two holes on each side of the channel to house four M12 thru bolts which are used in anchoring the steel channel to the concrete. The diameter of the holes is 14 mm to house the bolts. The centre of each hole is 20 mm away from each of its two sides and the spacing between two holes is 30 mm. Two types of bolts are tested in anchoring. The first type is called full-body stud bolt which passes through the whole width of the concrete beam. The other type of bolt is thru bolt which can be anchored to the concrete by drilling a hole to nearly half of the width of the beam and then pushing it into the hole using a hammer. In both cases, nuts are used to finish the anchoring.

### 2.3 Equipment setup and testing procedure

**Fig 5:**
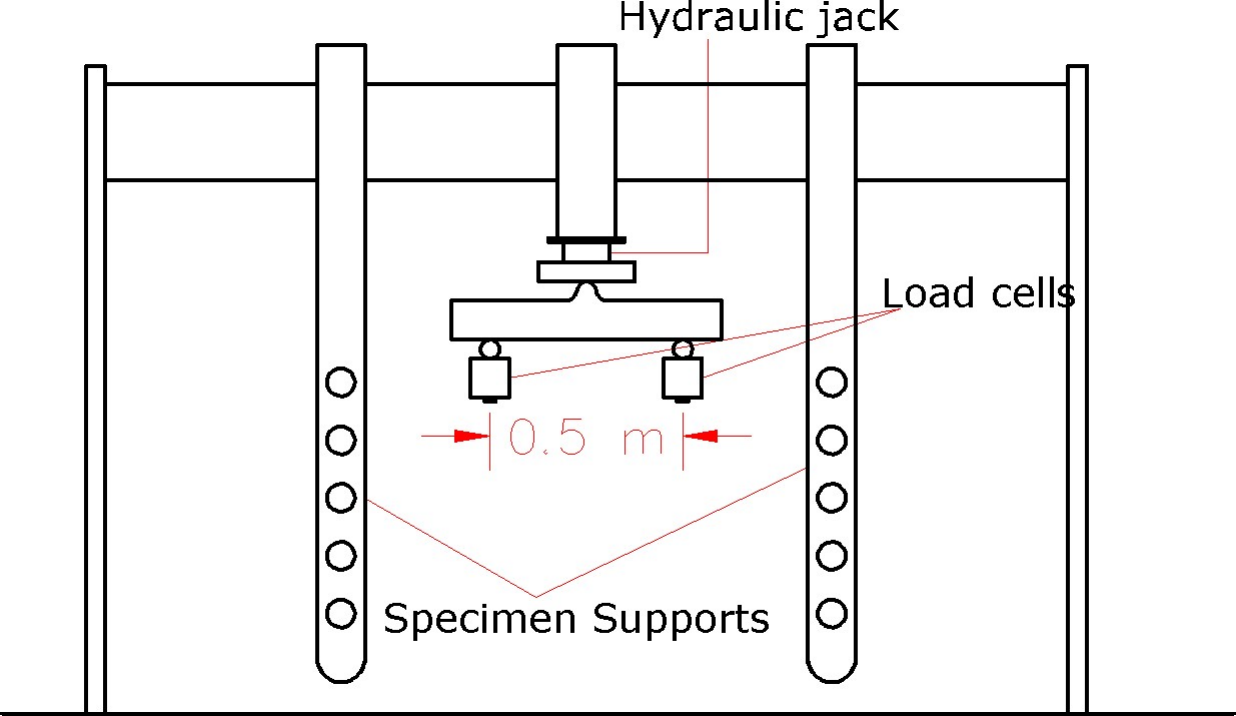
Diagram of the testing machine.

The testing machine has two movable supports and one optional support in the middle mounted on a steel frame and a jack fixed in the middle. The capacity of the jack is 80 tons with the rate of loading of approximately 0.3 mm/ min. Two load cells are used with a reading capacity of 30 tons each. Therefore, the written loads on the cracked beams in the Figures should be multiplied by two to find the total load- carrying capacity. A diagram of the testing machine is shown in Fig 5.

Four-point loading is used in all the tests to assure the bending failure in all the specimens as the aspect ratio of the distance between load and support to the depth of the beam is found to be 2.95 using Eq 1.


ad=19003240−25=2.95 (1)

To measure the strain inside the concrete, straps and the steel bars, two different types of strain gauges are used during the experiments. The first one is used on steel bars and metal straps. The length of this type is 30 mm. The length of the second type which is used on concrete is 80 mm. The length of LVDT used was 150 mm. A data logger is used to collect all the data from the load cells, strain gauges, and LVDTs.

## 3. Specimen preparation and description

### 3.1 Beam labels

The beam labels used to address them are shown in Table 6.

**Table 6:** Specimen labels in this paper.

Beam No.	Straps	Anchorage type	Distance to the edge (mm)	Extra Bolts to support	Edge Shape	Label
**76**	2 side 0 mm from bottom	2 Channels	350	No	Straight	76-F-2S0-CFSNB-350
**01**	2 side 0 mm from bottom	2 Channels	350	No	Round	01-F-2S0-CFRNB-350
**56**	2 side 0 mm from bottom	2 Channels	350	2 Bolts on each side	Round	56-F-2S0-CFRB-350
**75**	1 Bottom at center	2 Channels	350	No	Straight	75-F-1B-CWSNB-350
**101**	2 side 0 mm from bottom 1 bottom at the center	2 Channels	350	No	Straight	101-F-2S0-1BW-2CFWNB-350
**55**	2 side 0 mm from bottom	2 Channels	650 (800 mm c/c)	2 Bolts on each side	Round	55-F-2S0-2CFRB-650
**73**	2 straps at bottom	2 Channels	350	No	Round	73-F-2B-2CWRNB-350
**74**	Control					
**102**	Control					

**Fig 6:**
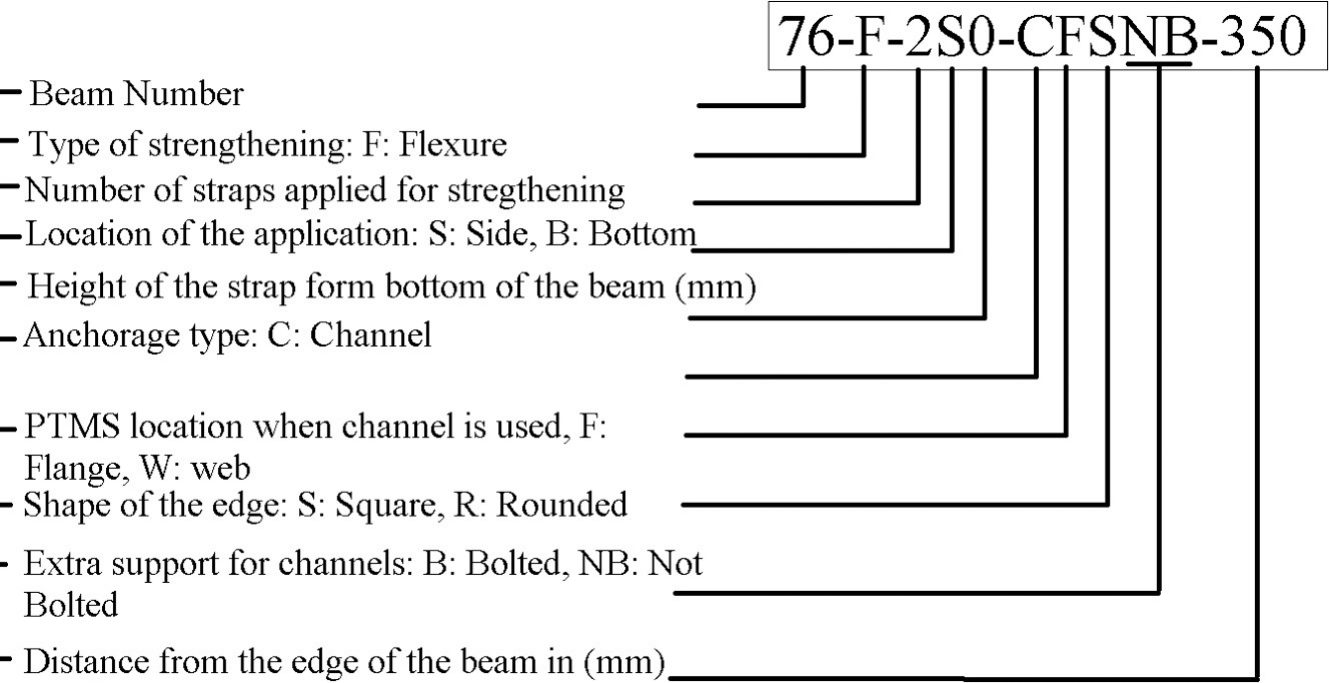
Beam label explanation.

The explanation of the labels is shown in Fig 6. For simplicity only beam number is used in the graphs and the tables.

### 3.2 Control specimens 74 and 102

The control specimen is designed to be tested without any strengthening.

The same configuration of the strain gauges is used in all the other specimens along with using other strain gauges to measure the strain inside the straps. The strain in the straps is measured after post tensioning, prior to testing, and during testing.

### 3.3 Concrete beam preparation for strengthening

**Fig 7:**
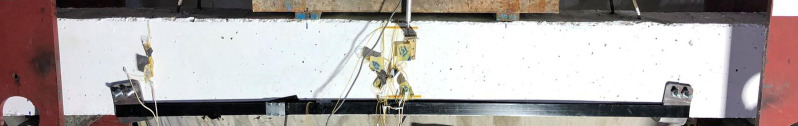
A slight change in the alignment of the channels.

The process of strengthening of the beams started by drilling holes to house the bolts which anchor the steel channels. For that, the location of the channels is marked based on the distance of the channel from the edge of the beam which varies between 350 mm and 650 mm. These distances are selected based on the absence of the cracks in the zone of 350 mm away from the edge of the beam and the presence of the stirrups in that area. This is confirmed by using a metal detector in that zone to find a location without a stirrup to avoid having a bolt passing through stirrups. The distance of 650 mm from the edge of the beam is chosen to block the flexural cracks in that area as the distance between the channels will be shorter which allows for more confinement from the straps that blocks the cracks more efficiently. The drilling of the holes takes place one by one and is double checked every time using the steel channel as a guide. After drilling the holes, the steel channels are set in place and the bolts are set in each hole using a hammer. Thru bolt of a length of 100 mm is tested against full body stud bolt. The authors found that thru bolt is better as it allows for drilling holes precisely without spalling in the concrete faces as only a certain depth of the hole is required. After drilling the holes, the steel channels are put in their locations and the four necessary thru bolts are anchored using a hammer to fix the channels. After fixing the channels, the straps are put in the slot between the concrete face of the beam and the face of the steel channel from right side of the beam. Then, the strap is pulled and put in the slot between the steel channel and the concrete face from the left side of the beam. After that the strap is wrapped around the channel from left side of the beam back to its other end on the right side of the beam. Then it is put inside the clip to be ready to apply tension on it and to be locked using notches. The pressure is applied on the straps in tensioning process until the pneumatic tensioner stopped tensioning. The existence of a fillet inside the channel due to bending process in making the channels and the 2 mm of tolerance of the holes to house the bolts allow the channels to change their vertical alignment and rotate slightly during post tensioning. This rotation works against the straps especially where it is wrapped around the web of the channel as during loading, it will be squeezed by the edge of the webs that bites into it and causes its rupture. It also creates a weak point for the straps applied on the flanges of the channel as it will create a concentrated point of pressure on the straps once they are wrapped around it. An example is shown in Fig 7. To prevent this a method is followed in this paper which is discussed in detail later.

In the following paragraphs, necessary procedures of strengthening for each specimen will be discussed.

#### 3.3.1 Beam 76 (76-F-2S0-CFSNB-350)

**Fig 8:**
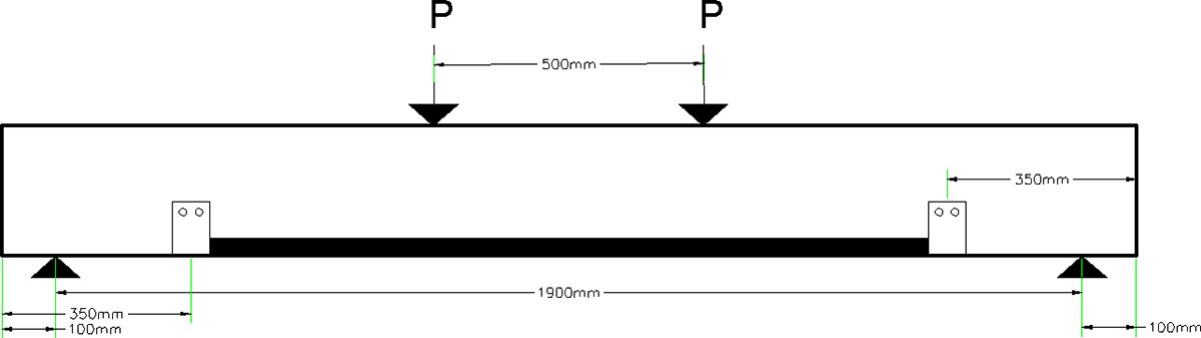
Beam 76 (76-F-2S0-CFSNB-350).

The beam is strengthened using two side straps wrapped around steel channel flanges from the front of the beam and the back of the beam. The steel channels are 350 mm away from the edges of the beam. The edges of the steel channels are square. The layout of the beam is shown in Fig 8.

#### 3.3.2 Beam 75 (75-F-1B-CWSNB-350)

**Fig 9:**
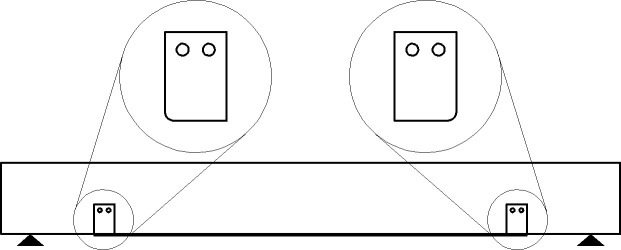
Rounded edge of the outer edge of the steel channels in beam 75.

This beam is strengthened using one strap wrapped around the web of the two steel channels located 350 mm away from the supports. The straps are applied in the middle of the channel web. The outer side of the channel web, where the straps are wrapped around it, is rounded to avoid having sharp ends that works like a weak point of the straps as shown in Fig 9.

#### 3.3.3 Beam 101 (101-F-2S0-1BW-2CFWNB-350)

**Fig 10:**
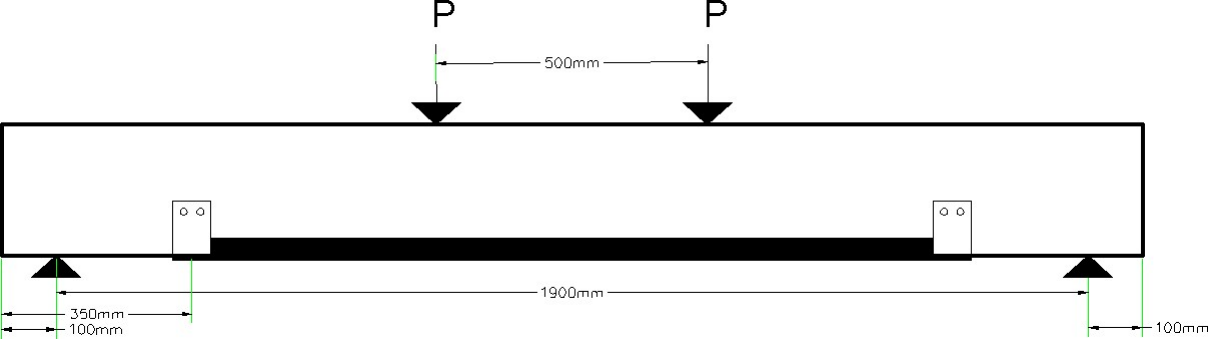
Beam 101 (101-F-2S0-1BW-2CFWNB-350).

The configuration of this beam is a combination of the two previous beams with rounding all the edges of the channel. So, the beam is strengthened using three straps, one at the bottom of the beam wrapped around the web of the channel and the other two on both sides of the beams wrapped around the flanges of the channels. The whole sides of the channel are rounded to avoid rupturing of the straps. The layout of the beam is shown in Fig 10.

#### 3.3.4 Beam 73 (73-F-2B-2CWRNB-350)

**Fig 11:**
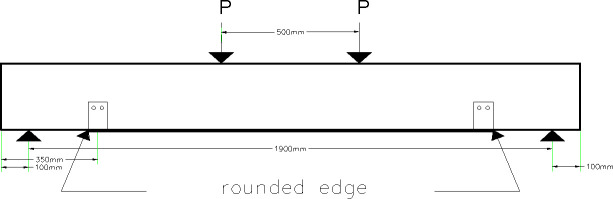
Beam 73 (73-F-2B-2CWRNB-350).

The configuration of this beam is exactly like beam 75 apart from using two straps at the bottom instead of one and rounding all the edges of the beam to avoid having any weak points in the steel channels for the straps. The layout of the beam is shown in Fig 11.

#### 3.3.5 Beam 56 (56-F-2S0-CFRB-350)

**Fig 12:**
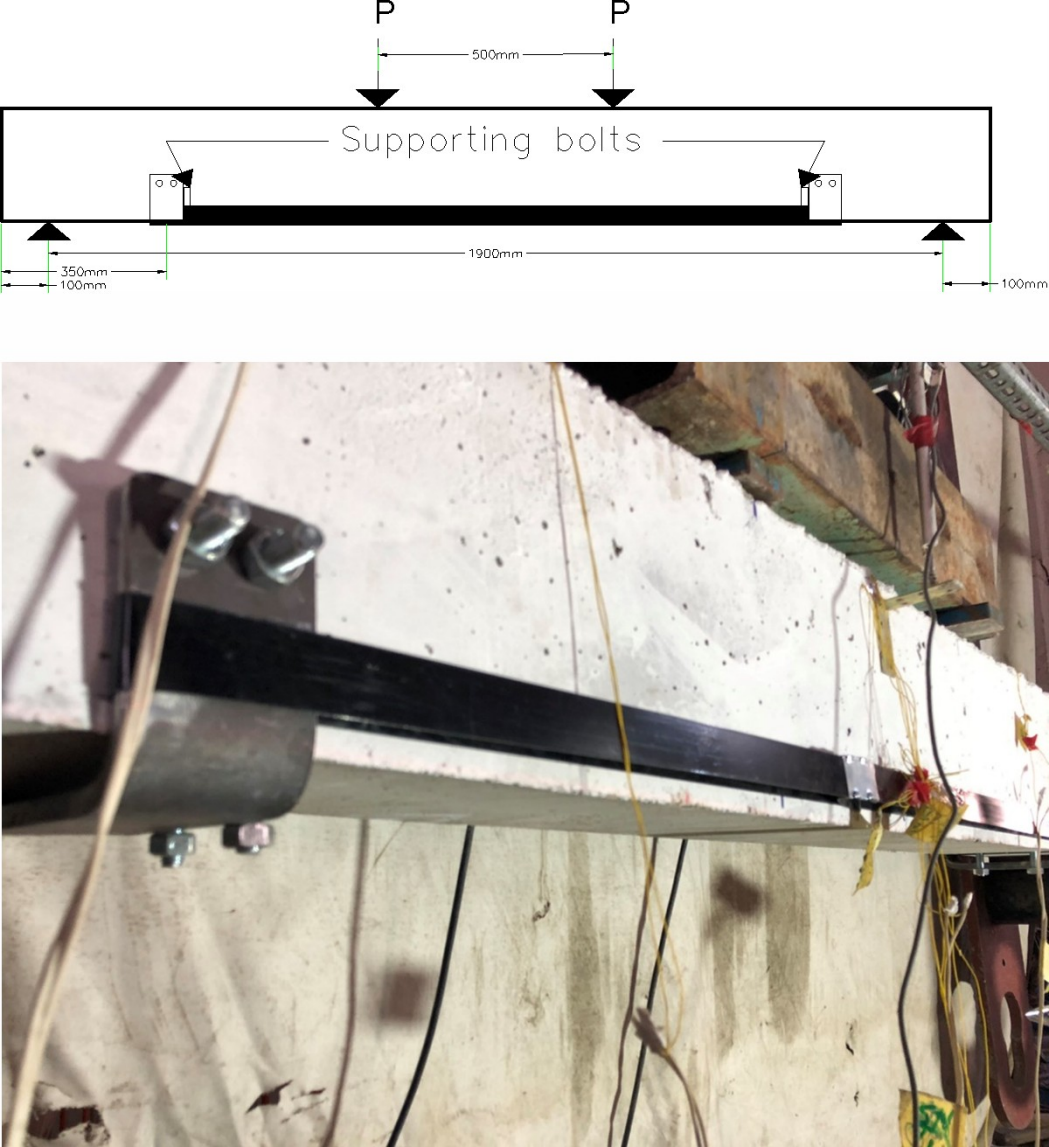
Thru bolts to support channels of beam 56. (a) Layout of the beam 56 with location of the supporting bolts. (b) Actual beam 56 with location of the supporting bolts.

The configuration of this beam is the same as beam 76, however, the edges of the channels are rounded using angle grinder to avoid creating weak points for the straps. Also, to have straight vertical alignment of the channel flanges, four thru bolts are used to support the channels at the bottom of the beam in a way that each channel is supported by two thru bolts. The bolts are driven into the bottom of the concrete in front of the channel web vertically as shown in Fig 12.

#### 3.3.6 Beam 55 (55-F-2S0-2CFRB-650)

**Fig 13:**
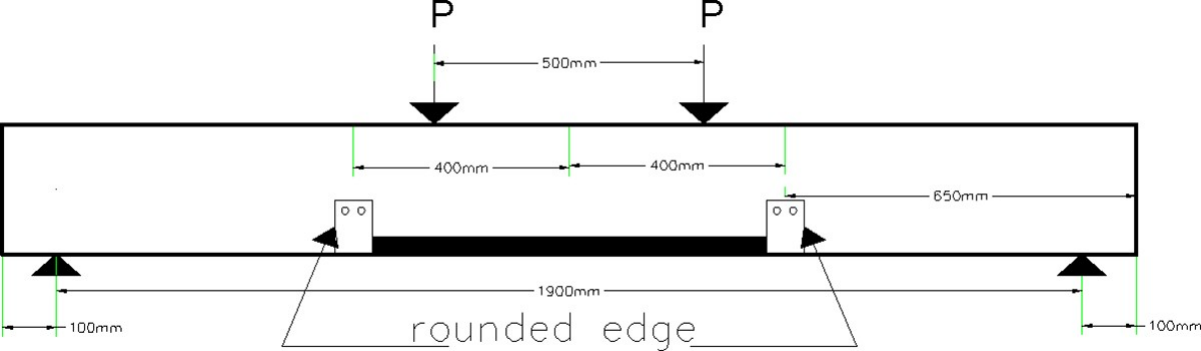
Beam 55 (55-F-2S0-2CFRB-650).

The main reason behind having this configuration is to limit the crack width in the area of the maximum bending. So, the beam is strengthened using only two straps wrapped around the flanges of two steel channels located at 650 mm from the edges of the beam. The edges of the channels are rounded. Also, two bolts are supporting each channel at the bottom of the beam to help the channel remain vertical. The layout of the beam is shown in Fig 13.

#### 3.3.7 Beam 01 (01-F-2S0-CFRNB-350)

**Fig 14:**
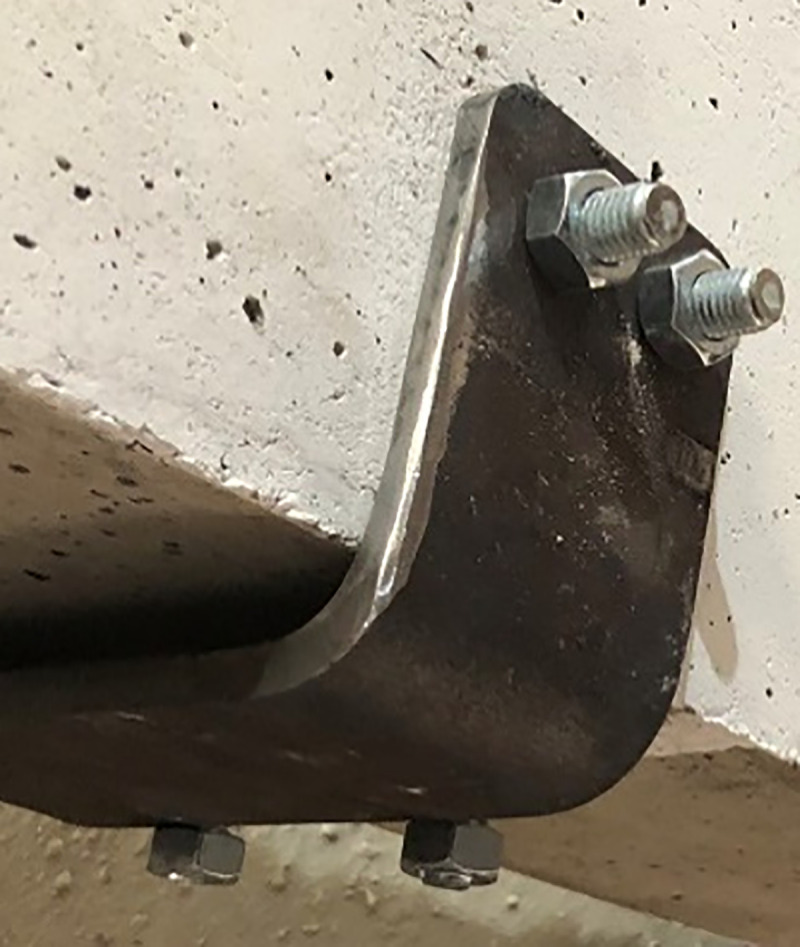
Fillet in the flanges of the channel.

The purpose is to find the effects of the edge shape of the channel on increasing the load carrying capacity of the beam. Therefore, the same configuration of beam 76 is repeated with rounding the edges of the steel channels only as shown in Fig 14. The vertical alignment of the channels is rotated slightly as there are no bolts supporting the web of the channel.

## 4. Results and discussion

The following results are achieved and discussed.

### 4.1 Selection of the bolt type

**Fig 15:**
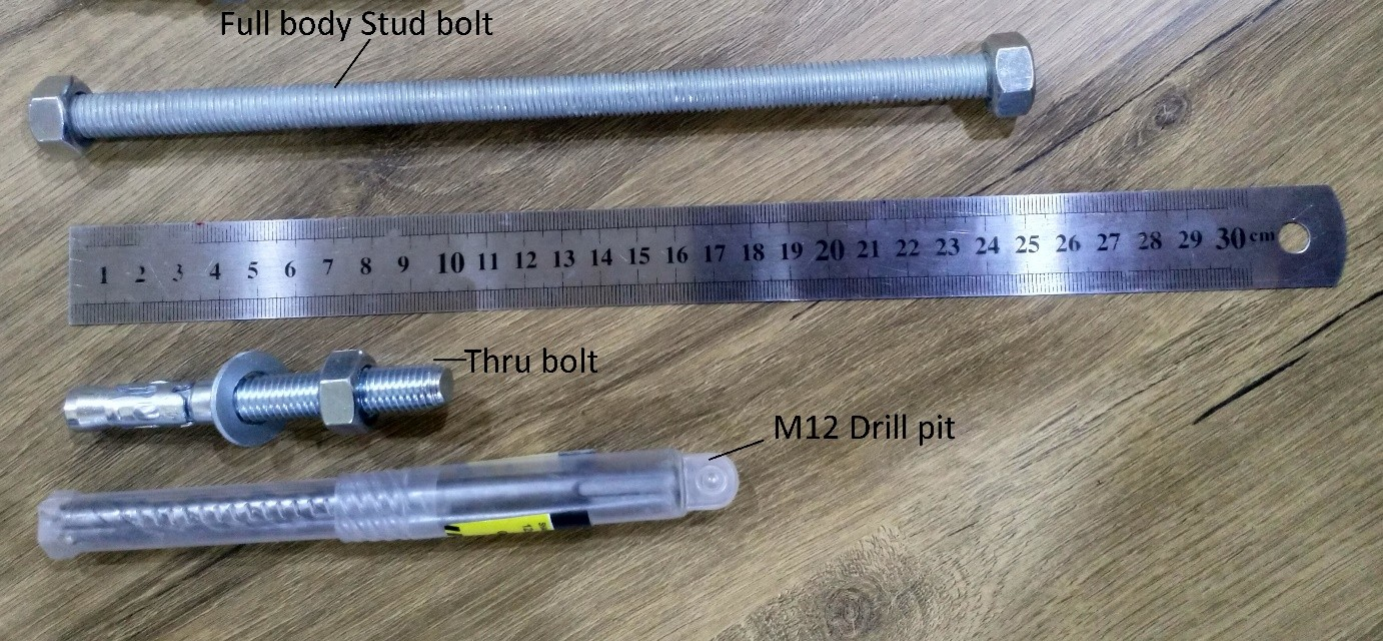
Bolt types tested with the drill pit used in drilling the concrete beam.

From the two types of the tested bolts, shown in Fig 15,which are full body stud bolt and thru bolt, it is proved that using thru bolts gives better results in anchoring the channels in an exact location. After testing both bolts on several normal R.C beams, it is proved that full body stud bolt cannot be used in practical even though theoretically it seems stronger than thru bolt. However, achieving holes drilled through a concrete beam with a drilling machine is nearly impossible. The reason is that path of the hole is changing during drilling making it impossible to create a linear hole from one exact point to another exact point through the beam. Also, drilling from one side to the other can cause spalling of the concrete which makes it lose some of its strength and creates a weak point on that face of the beam. On the other hand, using thru bolts of a length of 100 mm is tested giving accurate positions of the holes and it can successfully perform the job. The reason is that only a certain depth is required for the bolts then with a force of a hammer they can be locked and anchored in the concrete. Also, in using thru bolts, the holes are drilled separately which gives high accuracy and no spalling occurs during the process.

### 4.2 Comparison between beam specimens

#### 4.2.1 Load carrying capacity of all beams

**Fig 16:**
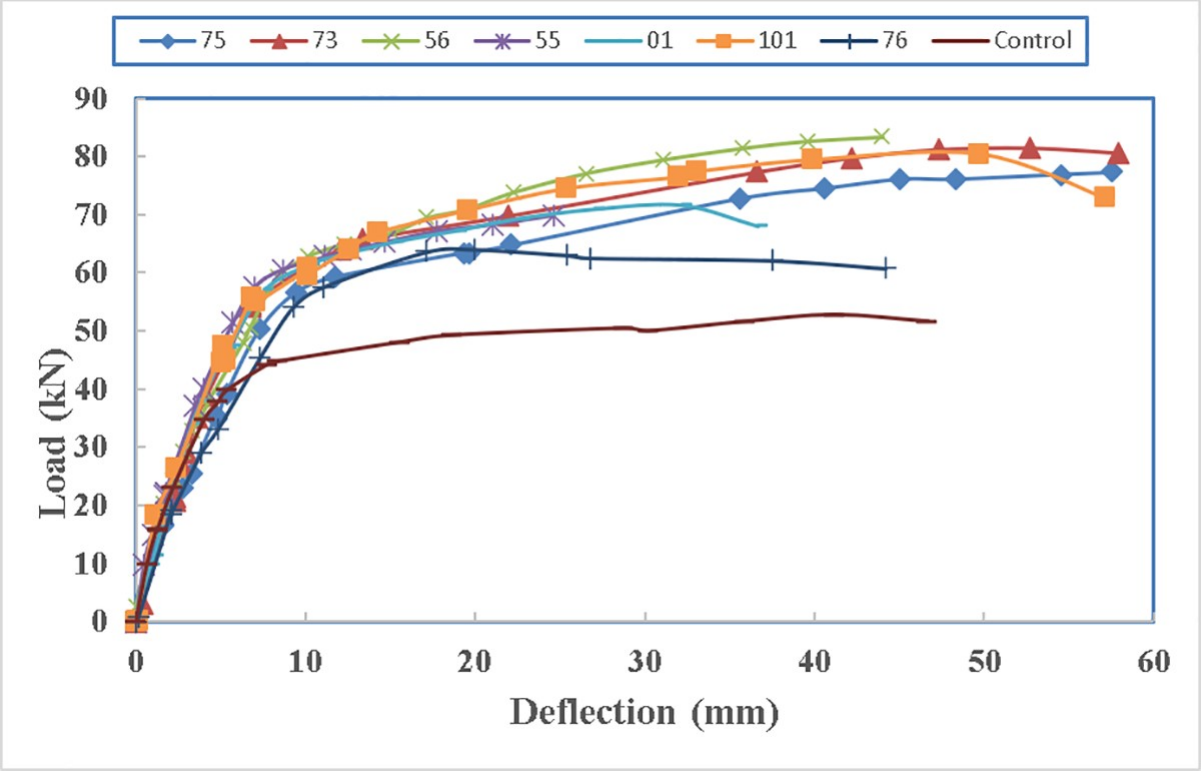
Load- deflection diagram of the beams.

In Fig 16, the load carrying capacity of the beams increases with providing straps wrapped around steel channel. The rate of increase varies between 29% for the beam 76-F-2S0-CFSNB-350 to 63% for the beam 56-F-2S0-CFRB-350. This is because beam 76 has no bolts to support the alignment of the channels and the channel edge is straight while beam 56 has bolts to supports its channels and the channel edges are rounded. Therefore, the key is to minimize the weak points and sharp edges for the straps as the straps are not the weak points. In terms of ductility, all the beams showed a ductile behaviour as they are designed for that purpose, however, their maximum limit of the deflection is not mentioned as the tests continued until collapse of the beam.

**Fig 17:**
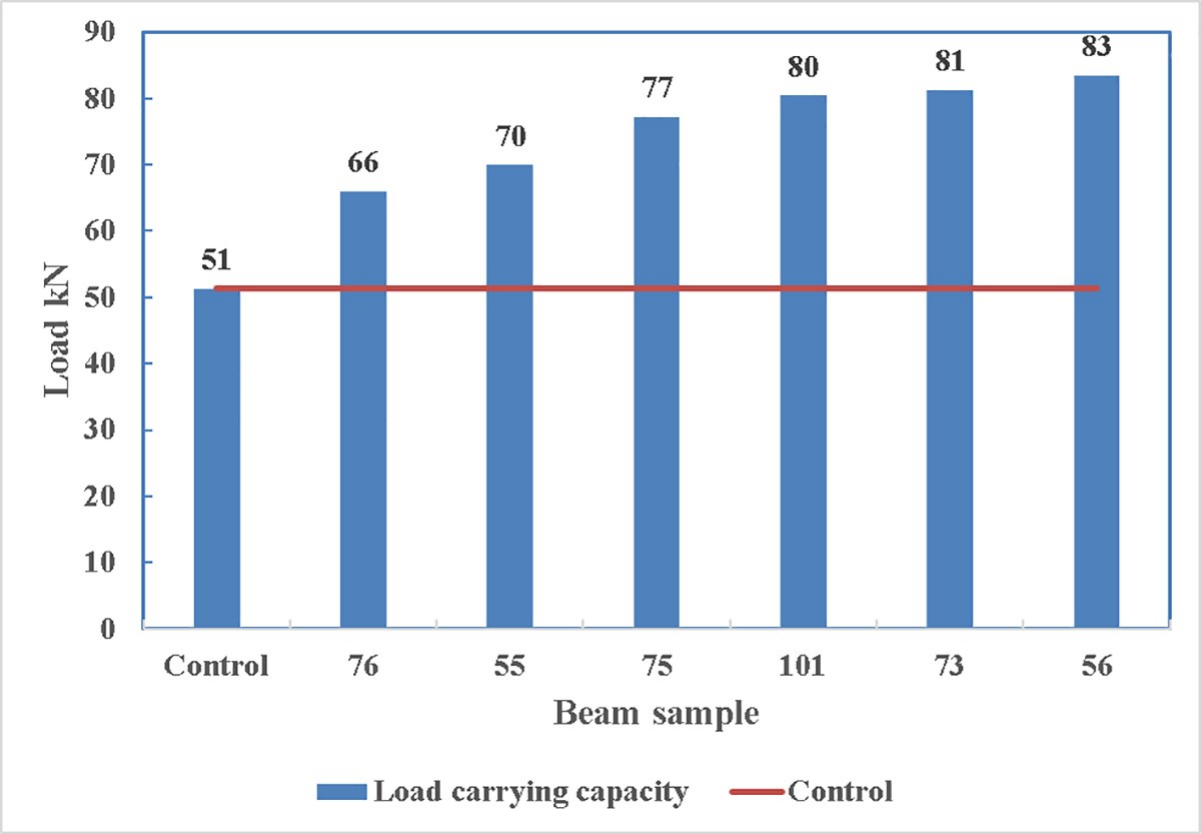
Comparison of load carrying capacity.

The load carrying capacity of the beams is shown in Fig 17 where all the other beams can carry more loads than the control beams. The maximum load being carried is by beam 56 with 83.4 kN and the minimum load is carried by beam 76 which is 66 kN.

#### 4.2.2 Failure mode of the beams

**Fig 18:**
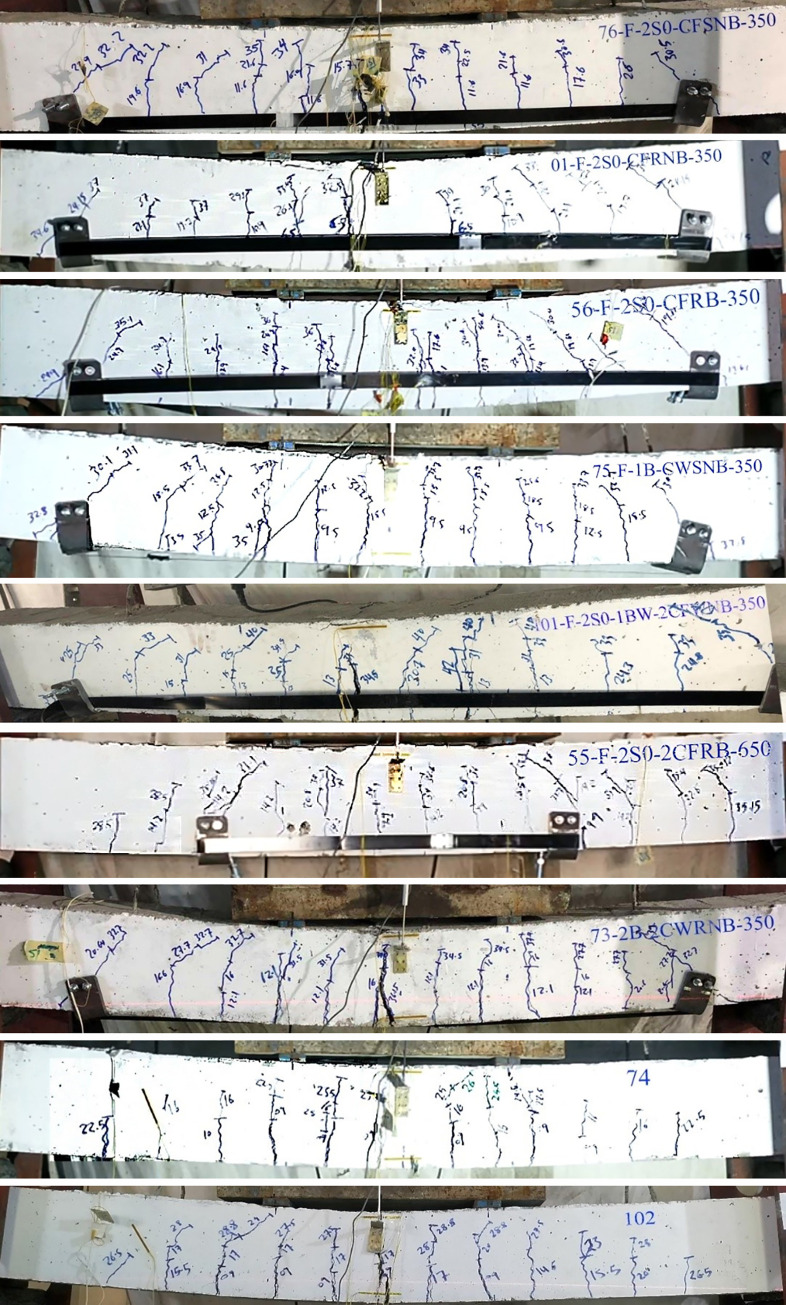
Crack pattern of the beams. (a) Crack pattern of beam No. 76. (b) Crack pattern of beam No. 01. (c) Crack pattern of beam No. 56. (d) Crack pattern of beam No. 75. (e) Crack pattern of beam No. 101. (f) Crack pattern of beam No. 55. (g) Crack pattern of beam No. 73. (h) Crack pattern of beam No. 74. (i) Crack pattern of beam No. 102.

The failure mode are shown in Table 7 and the crack pattern of the beams are shown in Fig 18A–18I.

**Table 7:** Failure mode and crack pattern of the beams.

Beam No.	Failure mode
**76**	Rupture of the strap
**01**	Concrete crushing in the middle
**56**	Rupture of the straps
**75**	Rupture of the strap
**101**	Rupture of the side straps and failure of the concrete
**55**	Crushing of the concrete around the anchored channel
**73**	Concrete crushing in the middle
**74**	Rupture of main steel bars
**102**	Rupture of main steel bars

#### 4.2.3 Effects of shape of the edge of the steel channels (beam 76 with two side straps on a squared edge flange vs beam 01 with two side straps on a rounded edge flange) and (beam 75 with one strap on the squared web of the beam vs beam 101 with one strap on the rounded edge web)

**Fig 19:**
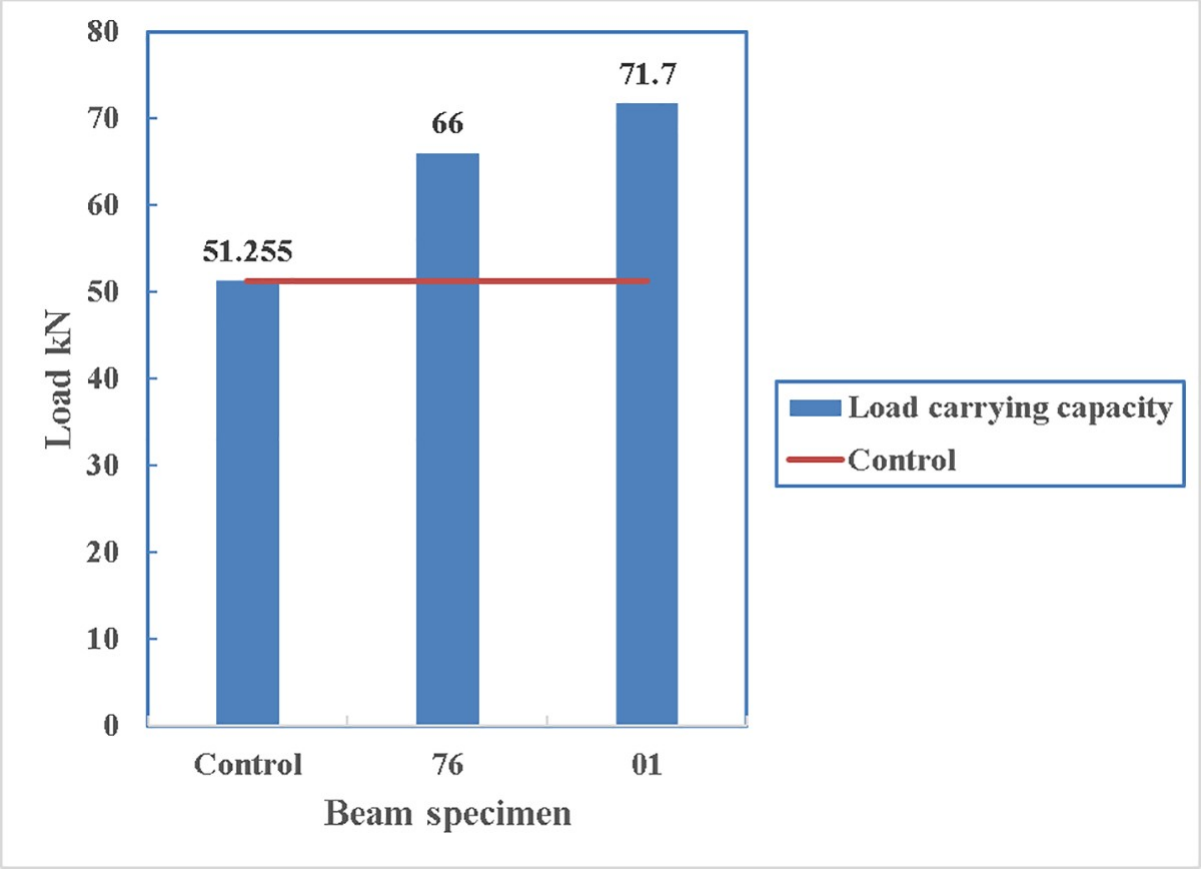
Comparison between beam 76 and beam 01.

In Fig 19, the load carrying capacity of the beam can increase by approximately 9% if the channel edges are rounded before applying the straps. This roundness can trigger the straps to work more efficiently as the strain develop in beam 01, before failure, is 500% more than that in the beam 76 with the straight edges. However, in both cases the load carrying capacity of the beam is more than that of the control specimen with the least of 29% for the beam 76 and 40% for the beam 01.

**Fig 20:**
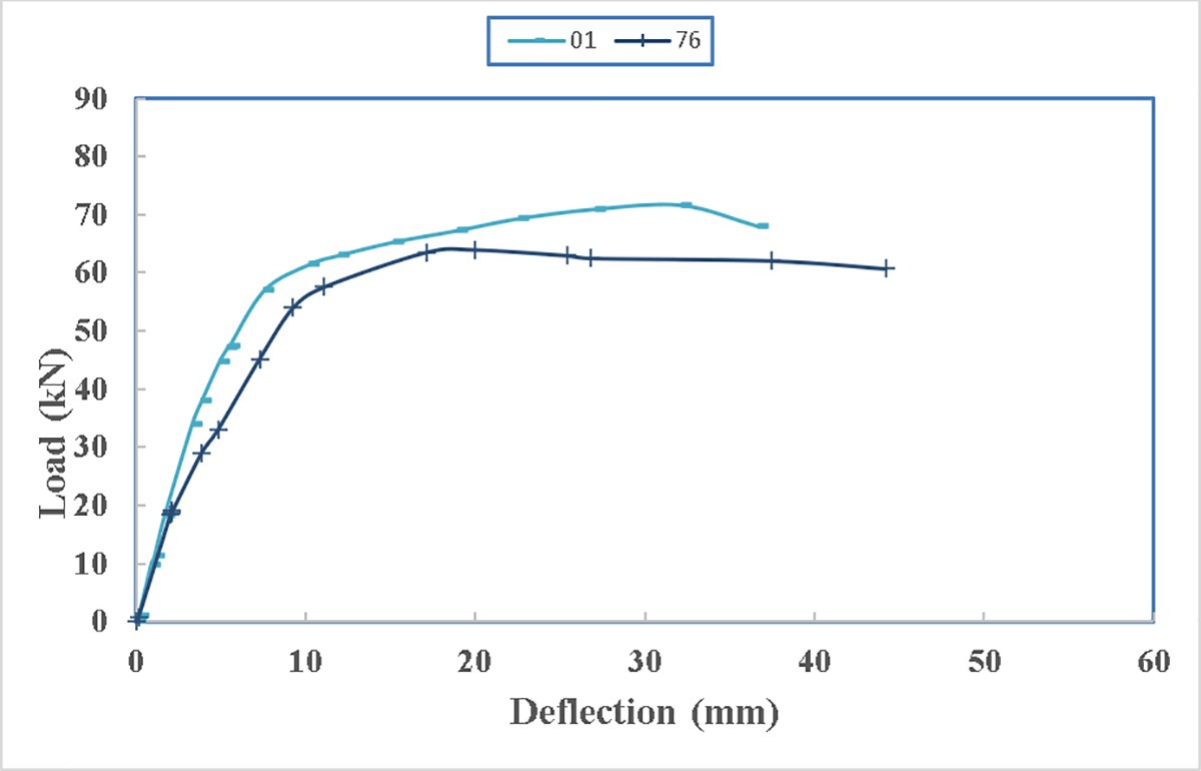
Load- deflection diagram of beams 76 and 01.

The load deflection diagram of both beams are shown in Fig 20. Beam 76 loses stiffness more compared to beam 01 due to insufficient confinement in the beam. Also, the beam loses strength after reaching its peak rapidly compared to beam 01 as it has been confined better due to smoothening the edges of the steel channel. Additionally, the deflection is more in the beam 76 as there was allowance for deflection due to the poor confinement provided by the straps.

**Fig 21:**
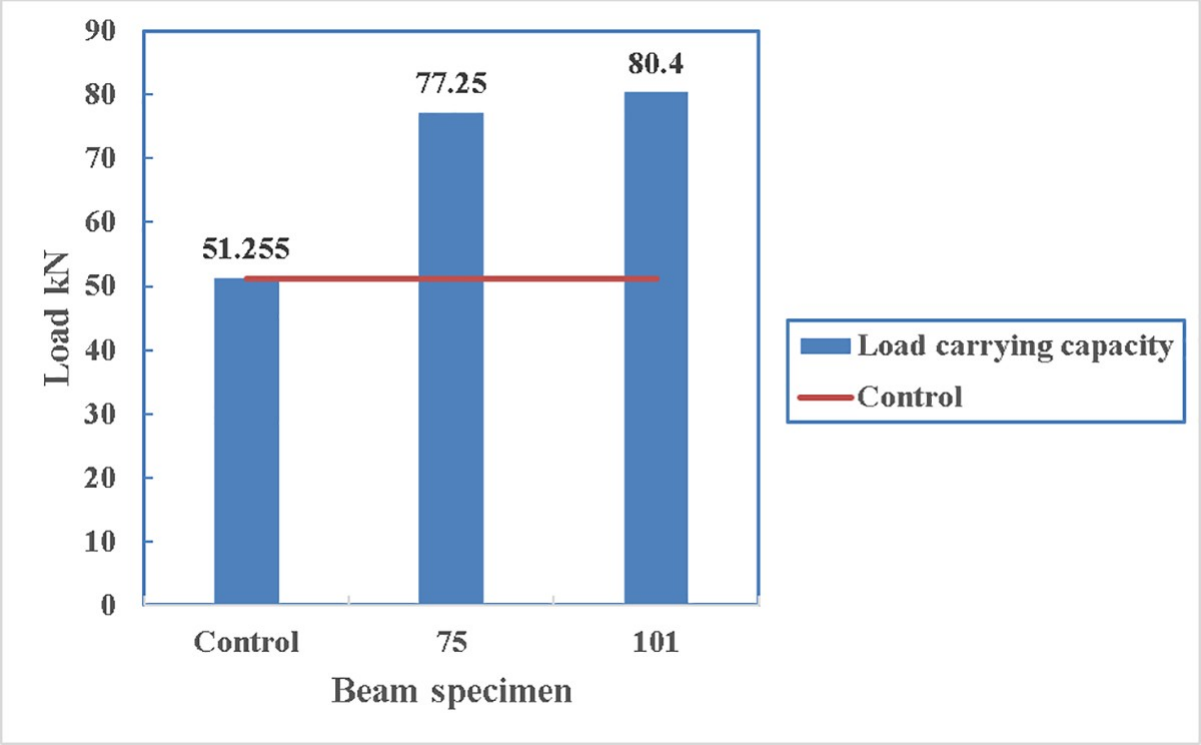
Load- carrying capacity of beams 75 and 101.

Similar phenomenon can be observed for the straps applied at the bottom of the beam. In Fig 21, the load carrying capacity is increased by only 4% by using rounded edge webs to house the straps. While that percentage was more for the straps applied on the flanges of the beam.

**Fig 22:**
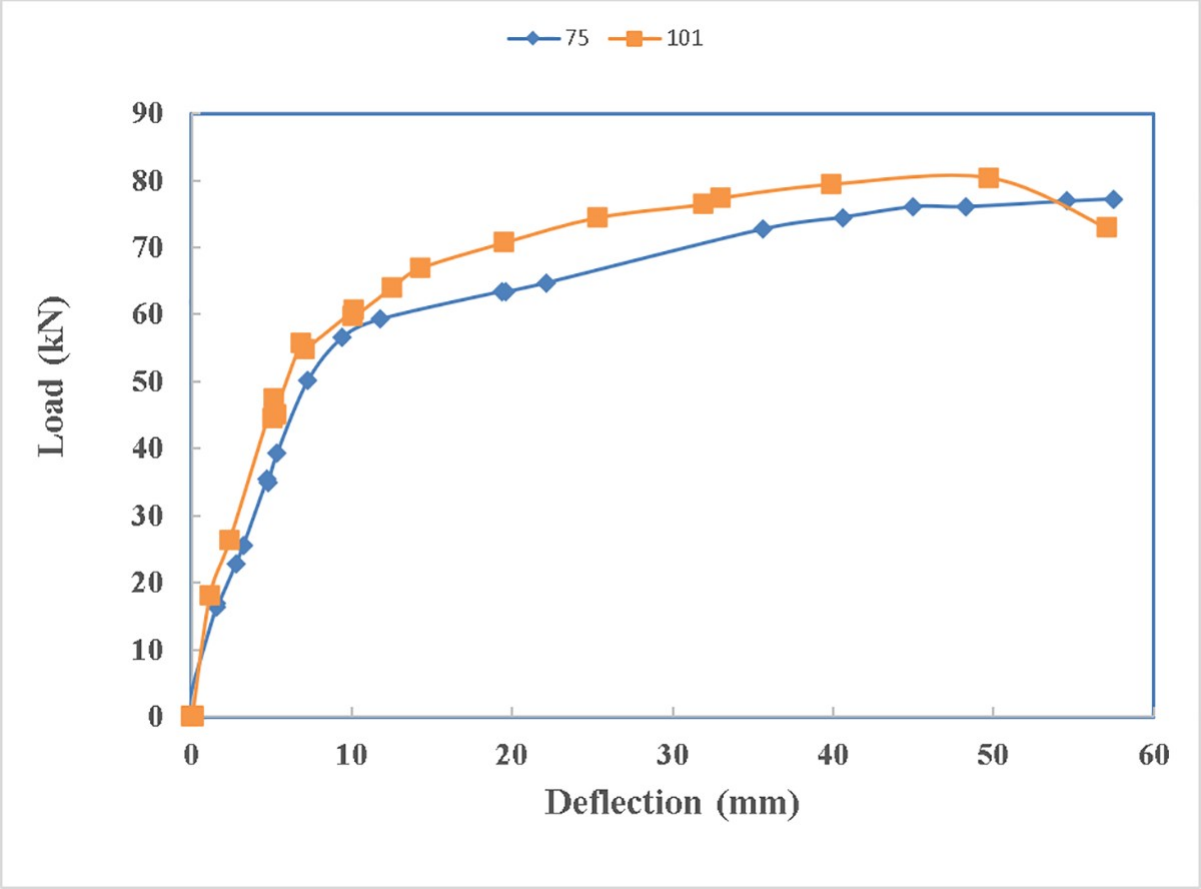
Load- deflection diagram of beams 75 and 101.

It is obvious from Fig 22, that beam 75 with squared edge webs started losing stiffness at earlier loads of 20 kN.

#### 4.2.4 Effects of the alignment of the steel channels (beam 01 vs beam 56)

**Fig 23:**
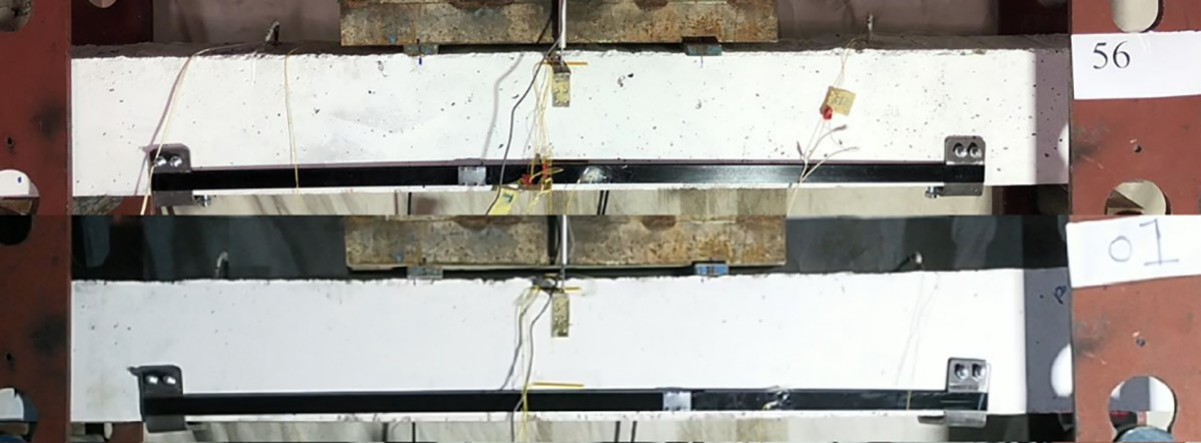
Vertical alignment of the channels of beam 56 and beam 01.

**Fig 24:**
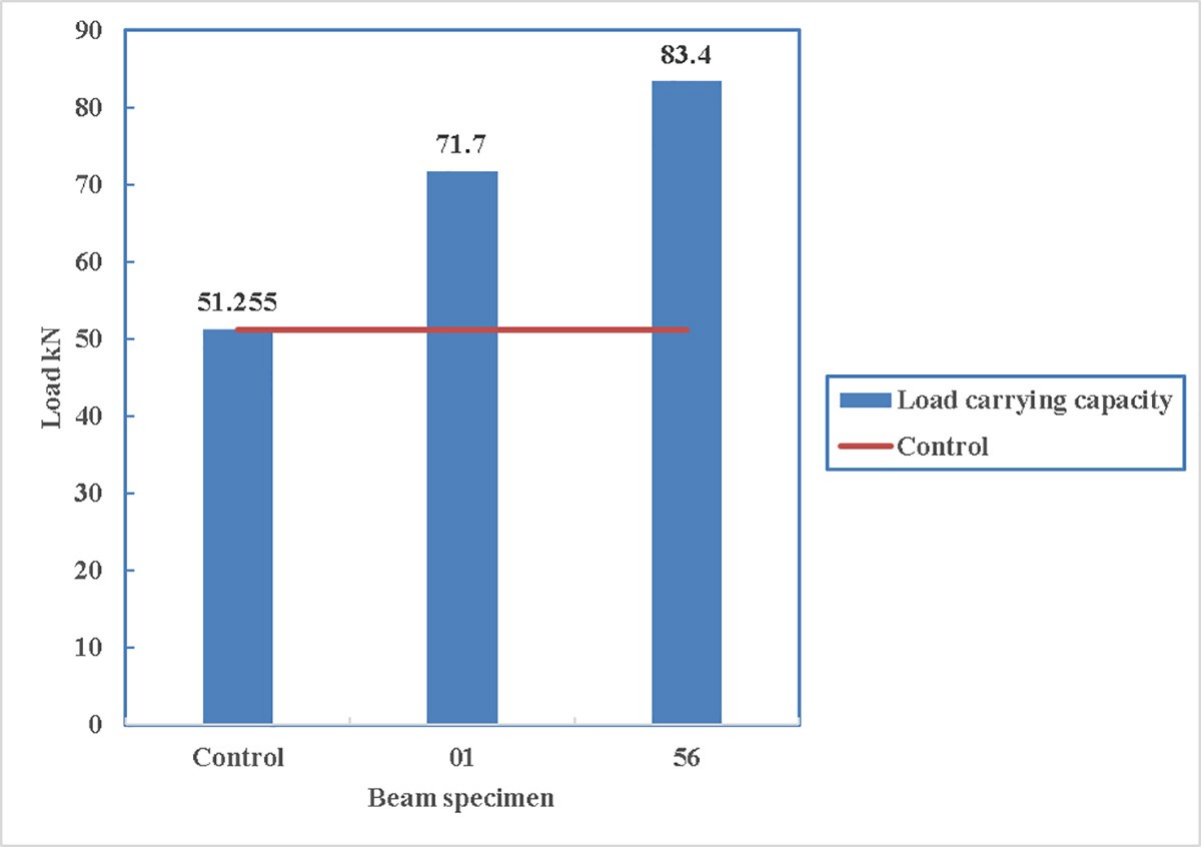
Effects of the anchorage type of steel channels between beam 56 and beam 01.

The other decisive factor in enhancing the load carrying capacity of the beam is the alignment of the flanges of the steel channels. This rotation is clear in Fig 23 where the flanges of beam 01 are rotated slightly inwards towards the centre of the beam compared to the flanges of beam 56 which is remained vertical. In Fig 24, that the load carrying capacity can increase by 16% if four thru bolts are provided at the bottom of the beam to support the flanges of the channels to remain vertical. However, in both cases the load carrying capacity increase substantially compared to the control specimens.

**Fig 25:**
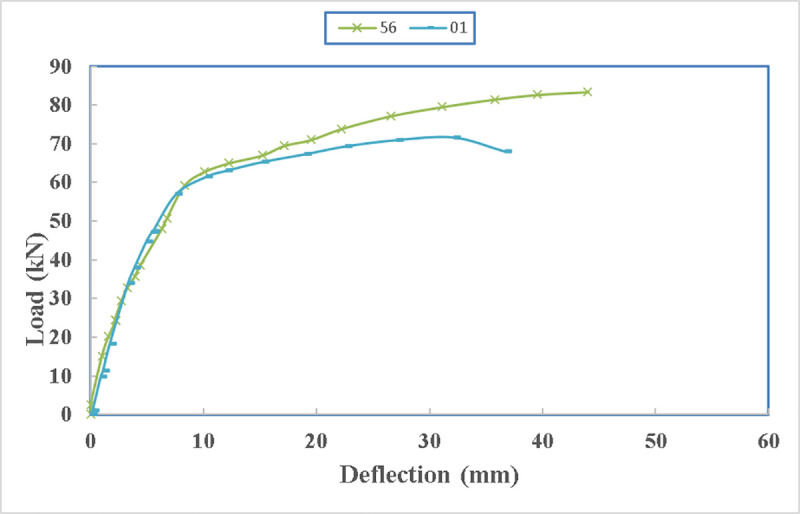
Load- deflection diagram between beam 56 and beam 01.

In Fig 25, the load- deflection diagram of the two beams are compared. Both beams have the same stiffness in the beginning showing that the initial confinement provided by the straps is the same. However, beam 01 which was not supported by extra bolts underneath the beam, so it lost its strength gradually while beam 56 gained more strength after load of 60 kN. Even after the ultimate load, beam 01 lost strength gradually due to spalling of the concrete at the anchorage location and punching the concrete from the steel channels. However, in beam 56 the beam reached its ultimate load carrying capacity but after the rupture of one of the straps it lost the strength rapidly. In beam 01 the straps remained unharmed while one of the straps in beam 56 ruptured at the maximum load. The reason for that rupture is a slight rotation of the edge as it is obvious from the data of the strain gauges. The strain developed in the ruptured strap is only 400 microstrain while the developed strain in the straps of beam 01 is 700 microstrain knowing that both straps were tensioned to develop approximately 200 microstrain in them during post tensioning process.

#### 4.2.5 Effects of the location of the anchorage for side strapping (beam 56 vs. beam 55)

**Fig 26:**
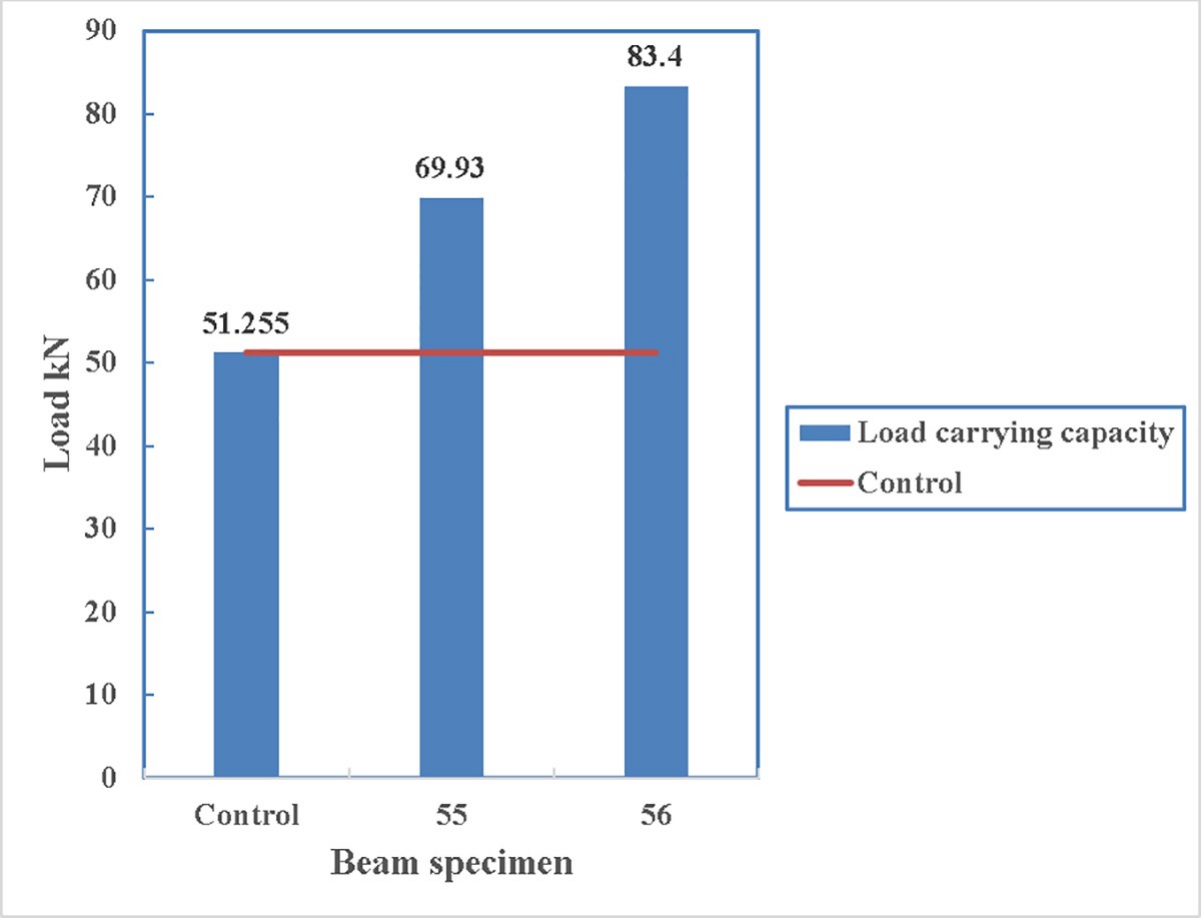
Load carrying capacity of beam 55 and beam 56.

Both beams have the same configuration but the location of the anchoring is different. Beam 56 is anchored with steel channels at 350 mm from beam edge while beam 55 is anchored with channels at 650 mm from the edge of the beam. The load carrying capacity of the beam decreases with an increase of the anchorage distance from the edge of the beams as shown in Fig 26. The load carrying capacity has decreased by 19% by shifting the anchorage to the bending zone by 300 mm. However, in both cases, the load carrying capacity increases compared to the control specimens. The crack width can be minimised by shifting the anchorage towards the bending zone which creates another issue due to the presence of the holes and a tensile force on the steel channels.

**Fig 27:**
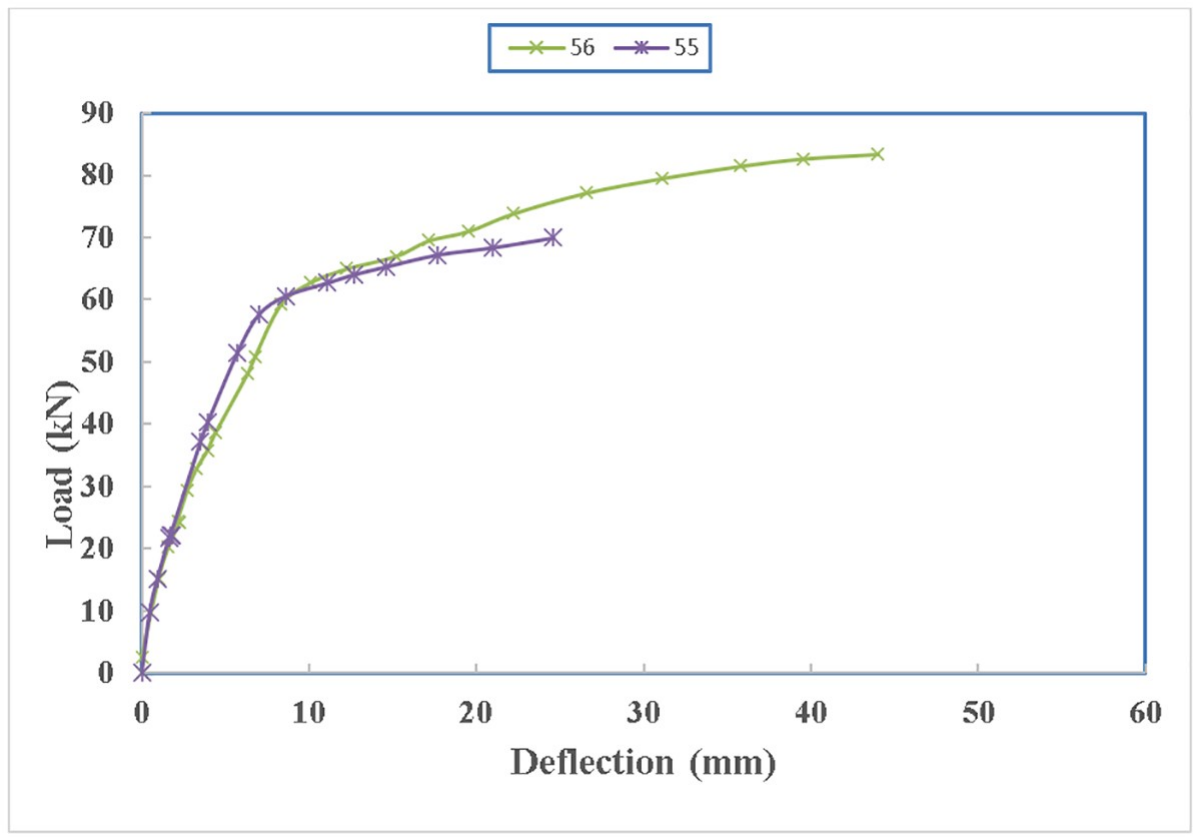
Load- deflection diagram between beam 55 and beam 56.

Both of the beams had the same stiffness as shown in Fig 27, however, beam 56 lost its stiffness earlier at a load of 35 kN pointing to the size of the cracks which were larger compared to the crack sizes of the beam 55. The deflection capacity of beam 56 is more than that of beam 55 even though, both straps in beam 55 were remained unharmed and one of the straps in beam 56 ruptured. This is due to having two anchored steel channels in the bending zone which curbed the ductility of the beam as the concrete spalled about the anchored channels.

#### 4.2.6 Effects of the number of straps at the bottom of the beam. (beam 101 with one strap vs. beam 73 with two straps)

**Fig 28:**
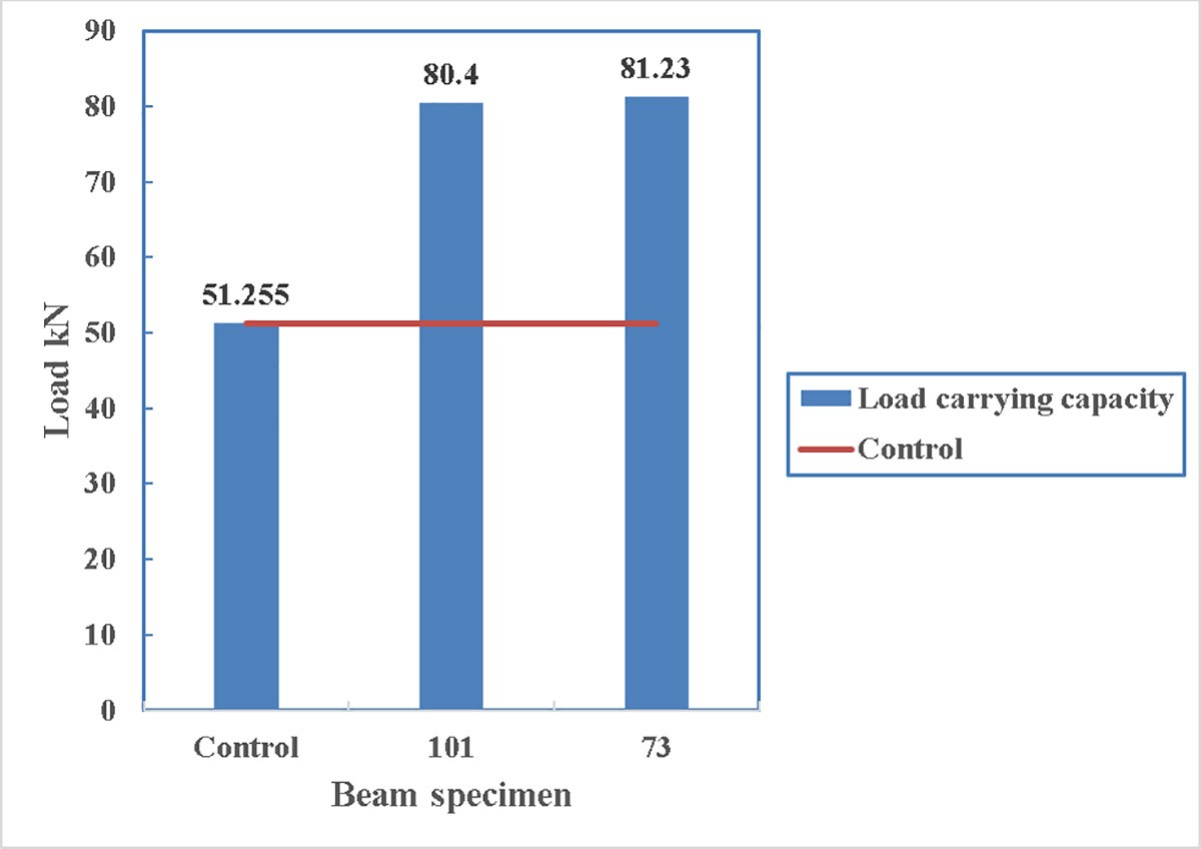
Load carrying capacity of beam 101 and beam 73.

The comparison is in between beam 101 which is technically strengthened using one strap at its bottom as the two side straps ruptured in the early stages of the loading and beam 73 which is strengthened using two straps at the bottom of the beam. The load carrying capacity can increase by only 1% if two straps are provided instead of one as shown in Fig 28. The reason for it, is that even if only one strap is provided, the maximum strength of the strap cannot be reached as the concrete fail prior to that. Both beams have strengths more than that of the control specimen.

**Fig 29:**
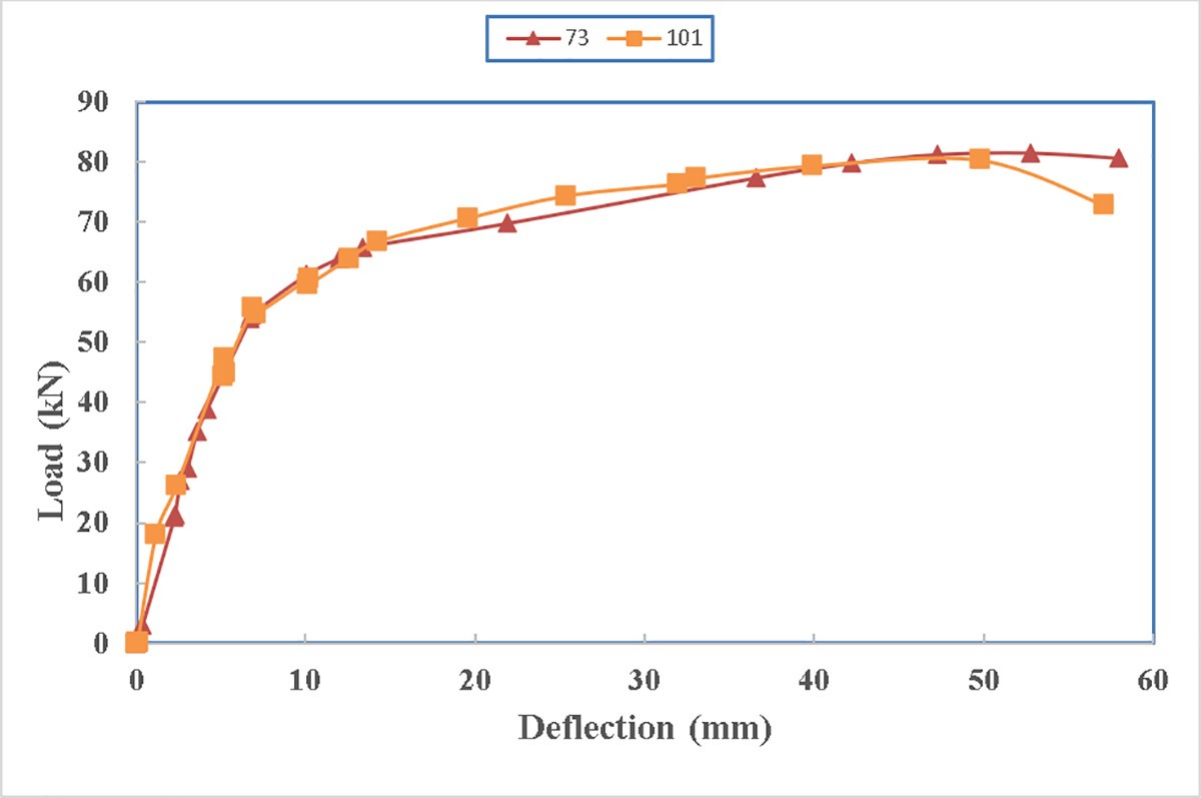
Load- deflection diagram between beam 73 and beam 101.

The load carrying capacity of the beams, shown in Fig 29, are similar. The small fluctuation in the stiffness of beam 73 can be due to energy dissipation from the cracks in the beam section and then regaining the stiffness due to the confinement.

#### 4.2.7 Effects of the location of the strap application (beam 75 with one strap at the bottom vs beam 76 with two of side straps)

**Fig 30:**
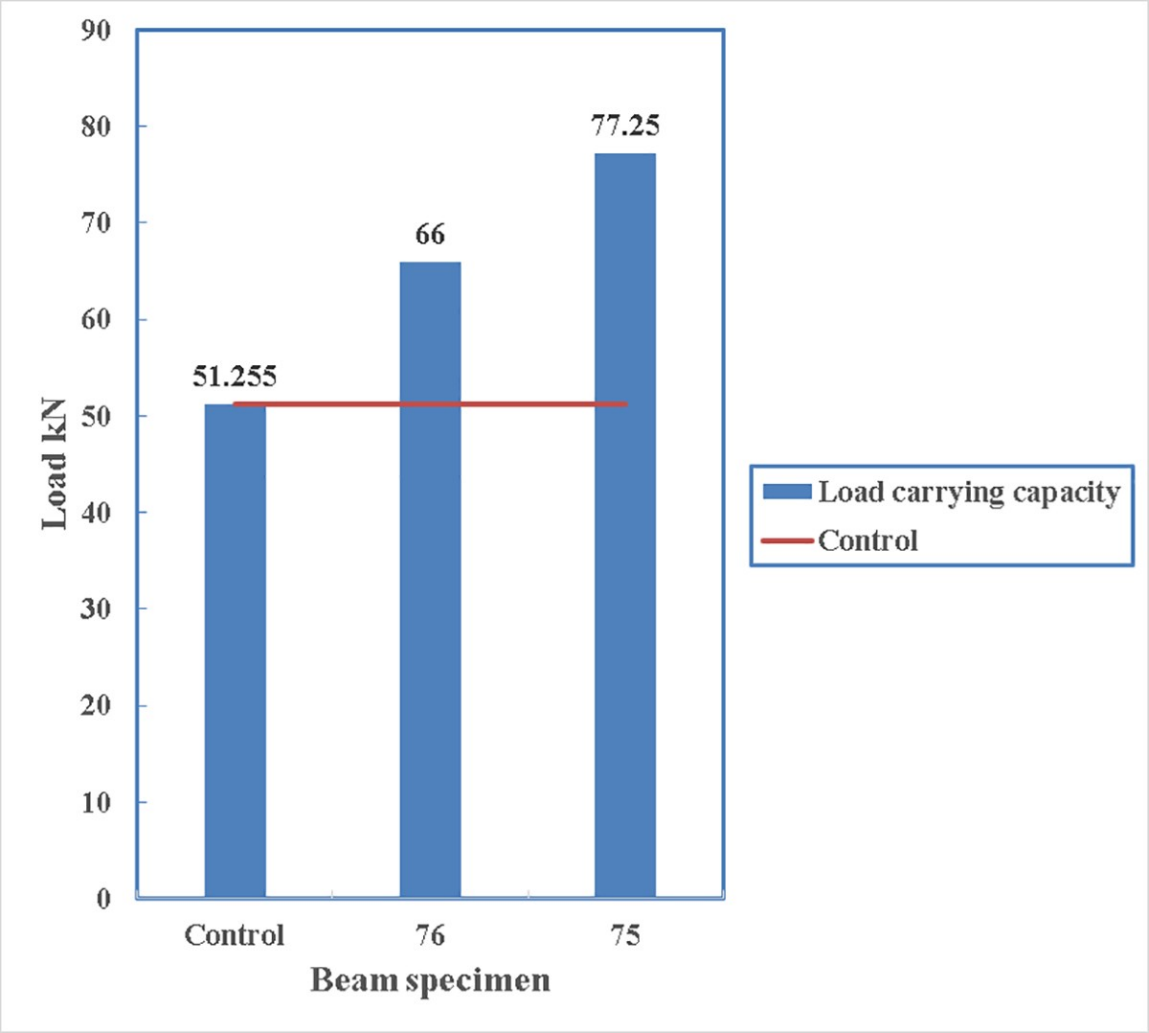
Load carrying capacity of beam 75 and beam 76.

As the resisting moment of a beam is the tensile force of the beam times the lever arm to the centre of the compression block therefore, it is logical to have more load carrying capacity if the straps are applied at the lowest point of the beam. In Fig 30, the load carrying capacity of the beam with straps at the bottom is 77.25 kN while it is only 66 kN for the beam with two side straps. This increase is 17%. This confirms the idea of having strengthening at the bottom of the beam rather than the sides.

**Fig 31:**
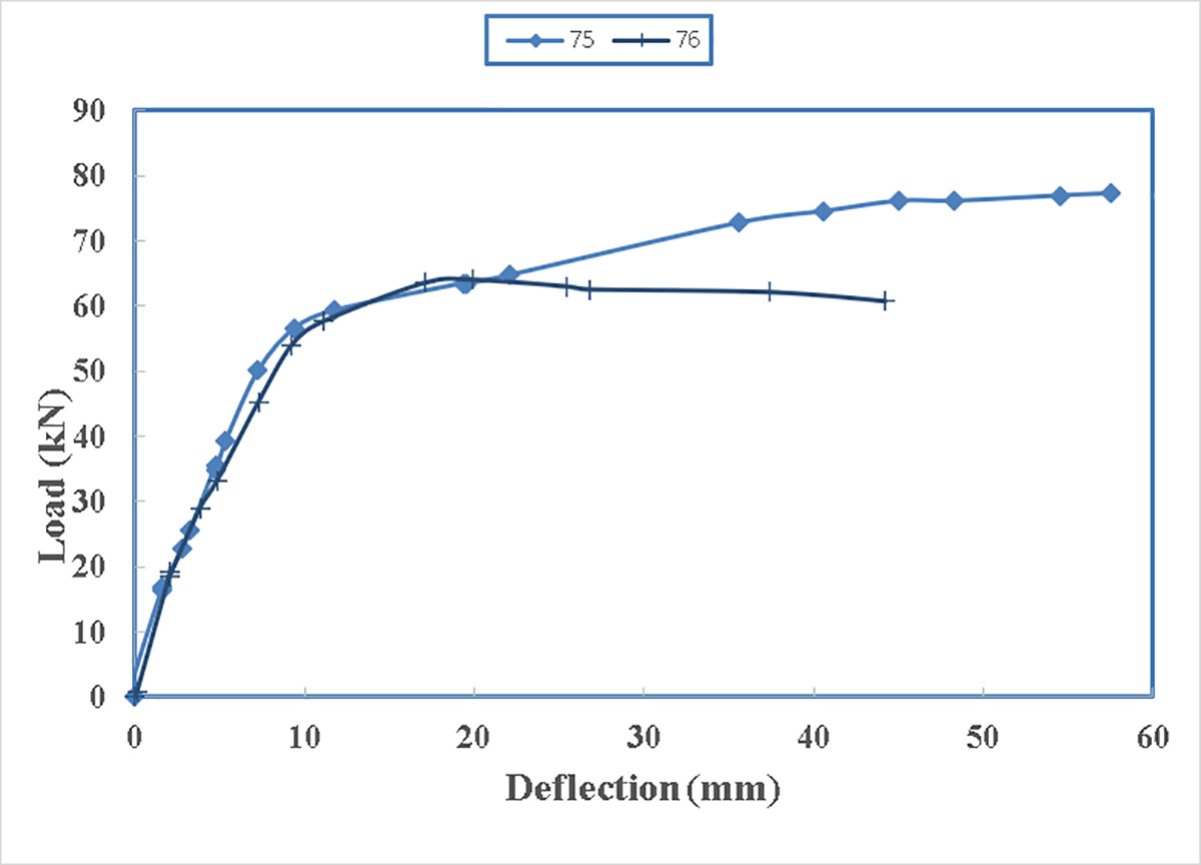
Load- deflection diagram between beam 75 and beam 76.

Their load-deflection diagram, shown in Fig 31, is similar until the load of 66 kN where beam 76 reaches its maximum capacity. Then beam 76 with two straps loses strength gradually. On contrary, beam 75 gains strength until it reaches its maximum load carrying capacity of 77.25 kN.

## 5. Conclusions

This paper investigated the structural behaviour of normal concrete beams strengthened using post-tension metal straps (PTMS) wrapped around anchored steel channels in the bending area.

1. Normal R.C beams with a/d ratio of 2.95 will fail in bending in a ductile failure, however, strengthening can be performed in terms of increasing the load carrying capacity of the beam.
2. It is proven that using PTMS can increase the load carrying capacity of normal R.C beams when wrapped around steel channels. The rate of increase can vary between 29% to 63%.
3. Using thru bolts is better than full body stud bolts in anchoring the channels as precision is required in the location of the holes for anchoring the channels.
4. The most effective factor contributing in enhancing the load carrying capacity in beams strengthened with PTMS wrapped around the web of the channel is the roundness of the webs of the channels. The load carrying capacity can increase by 4% due rounding the web edges. The effect is substantial in case of wrapping the straps around the flanges of the channel where there is an increase of 9% in load carrying capacity if the edges of the channel are rounded.
5. However, the most effective factor in increasing the load carrying capacity in beams strengthened with straps wrapped around the flanges of the channel is the alignment of the flanges. The load carrying capacity of the beam can increase by more than 16% if the alignment of the flanges is kept vertical by using thru bolts to support the channels.
6. It is proven that applying straps at the bottom of the beam is more effective than applying them on the side of the channels regardless of the number of the straps. This increase is approximately 17%.
7. The number of straps applied at the bottom of the beam cannot affect the load carrying capacity of the beam if applied on a rounded-web channel.
8. The location of the steel channels anchored to the beam influences the total load carrying capacity of the beam. Even though it minimizes the size of the cracks if the channels are applied close to the bending zone of the beams, however, they create weak points for the concrete to spall. Therefore, the location of the anchored steel channels is recommended to be close to the supports.
9. The crack width can be reduced and localized to the tension zone of the beam once the beam is strengthened with PTMS around anchored steel channels at 650 mm from the edge of the beam. However, concrete might spall around the anchorage area.
10. The strain developed in the straps during post-tensioning changes with the distance between the anchored steel channels. The more that distance is, the less developed strain would be in the metal straps. Therefore, to get better confinement the distance between the steel channels should be minimized.

## Supporting information

S1 Data (XLSX)
